# Coral lipid bodies as the relay center interconnecting diel-dependent lipidomic changes in different cellular compartments

**DOI:** 10.1038/s41598-017-02722-z

**Published:** 2017-06-12

**Authors:** Hung-Kai Chen, Li-Hsueh Wang, Wan-Nan U. Chen, Anderson B. Mayfield, Oren Levy, Chan-Shing Lin, Chii-Shiarng Chen

**Affiliations:** 1 Department of Marine Biotechnology and Resources, National Sun Yat-sen University, Kaohsiung, 804 Taiwan; 2 Graduate Institute of Marine Biology, National Dong-Hwa University, Checheng, Pingtung 944 Taiwan; 3 Taiwan Coral Research Center, National Museum of Marine Biology and Aquarium, Checheng, Pingtung 944 Taiwan; 4 Department of Biological Science and Technology, I-Shou University, Kaohsiung, 824 Taiwan; 5 Khaled bin Sultan Living Oceans Foundation, Annapolis, MD 21403 United States of America; 6 The Mina and Everard Goodman Faculty of Life Sciences, Bar Ilan University, Ramat Gan, 52900 Israel

## Abstract

Lipid bodies (LBs) in the coral gastrodermal tissues are key organelles in the regulation of endosymbiosis and exhibit a diel rhythmicity. Using the scleractinian *Euphyllia glabrescens* collected across the diel cycle, we observed temporally dynamic lipid profiles in three cellular compartments: host coral gastrodermal cells, LBs, and *in hospite Symbiodinium*. Particularly, the lipidome varied over time, demonstrating the temporally variable nature of the coral–*Symbiodinium* endosymbiosis. The lipidome-scale data highlight the dynamic, light-driven metabolism of such associations and reveal that LBs are not only lipid storage organelles but also act as a relay center in metabolic trafficking. Furthermore, lipogenesis in LBs is significantly regulated by coral hosts and the lipid metabolites within holobionts featured predominantly triacylglycerols, sterol esters, and free fatty acids. Given these findings through a time-varied lipidome status, the present study provided valuable insights likely to be crucial to understand the cellular biology of the coral–*Symbiodinium* endosymbiosis.

## Introduction

The coral–dinoflagellate endosymbiosis is generally defined as mutualistic because both partners benefit from the relationship^1^. Coral hosts provide certain carbon and nitrogen skeletons for the photosynthetic reactions of *Symbiodinium*, and *Symbiodinium* translocate most of their photosynthetically fixed carbon, including glycerol, glucose, amino acids, and lipids, to the coral host^2–4^. *Symbiodinium* may incorporate various lipids, sugars, and amino acids into the host cells or tissues^5, 6^. Irrespective of the species of *Symbiodinium*, translocated metabolites affect the host lipid levels, thereby affecting reproduction, growth, and ultimately fitness of the host^7, 8^. Lipids are the primary long-term source of stored energy in corals, and several studies have indicated that lipids play a pivotal role during endosymbiosis regulation^9, 10^. Moreover, fatty acid profiles, including the concentration and composition of the saturated fatty acid (SFAs) and polyunsaturated fatty acids (PUFAs) pools, are reflective of lipid biogenesis *in hospite* and may be used to highlight lipid flux within the holobiont (host and endosymbionts)^11^.

During endosymbiosis, various lipids such as wax esters (WEs), triacylglycerides (TAGs), sterol esters (SEs), cholesterols (Cols), phospholipids (PLs), and free fatty acids (FFAs) accumulate within organelle-like structures known as LBs^12–16^. From archaea to mammals, one of the main functions of LBs is intracellular trafficking of lipids, thereby emphasizing their critical role in cell biology^17, 18^. To date, the functional formation of coral LBs during endosymbiosis remains to be elucidated^16^. The relationship between LB formation and the regulation of the coral–dinoflagellate endosymbiosis is evidently quite intimate, and studies describing the cellular mechanisms underlying the regulation of lipid biogenesis have been mostly cursory and preliminary. Previous studies on protein sequencing data and microscopic observations suggest that both *Symbiodinium* and host organelles are involved in LB biogenesis^13, 19^. Furthermore, Luo *et al*. and Chen *et al*. have demonstrated that coral endosymbiotic status positively correlated with symbiont population and variable changes of LBs, and implied that endosymbiosis may be dynamic^12, 14^.

These studies also revealed that dynamic LBs may highly correlate with potentially critical consequences for symbiosis function. In particular, coral gastrodermal LBs change in density, morphology, composition, and intracellular distribution over diel cycles^14^. However, no study has investigated diel lipidomic patterns of various cellular compartments in corals to determine the relative importance of diel changes. Therefore, “time and composition” information regarding LB biogenesis within the host cell cytoplasm is crucial. Because coral metabolism is easily influenced by light or photosynthesis^20^, it was particularly hypothesized that the lipidomes within different cellular compartments vary over diel cycles. The present study investigating the temporal lipidome of LB biogenesis, metabolism, and trafficking within the coral host–*Symbiodinium* association across the diel cycle may provide an insight into endosymbiotic regulation. We hope that by shedding light on the lipid composition of these cellular fractions at various time scales, a more efficiently defined working model for intra-holobiont lipid trafficking can be established.

## Results

### Diel fluctuation of lipid and fatty acid concentrations

**Figure 1:**
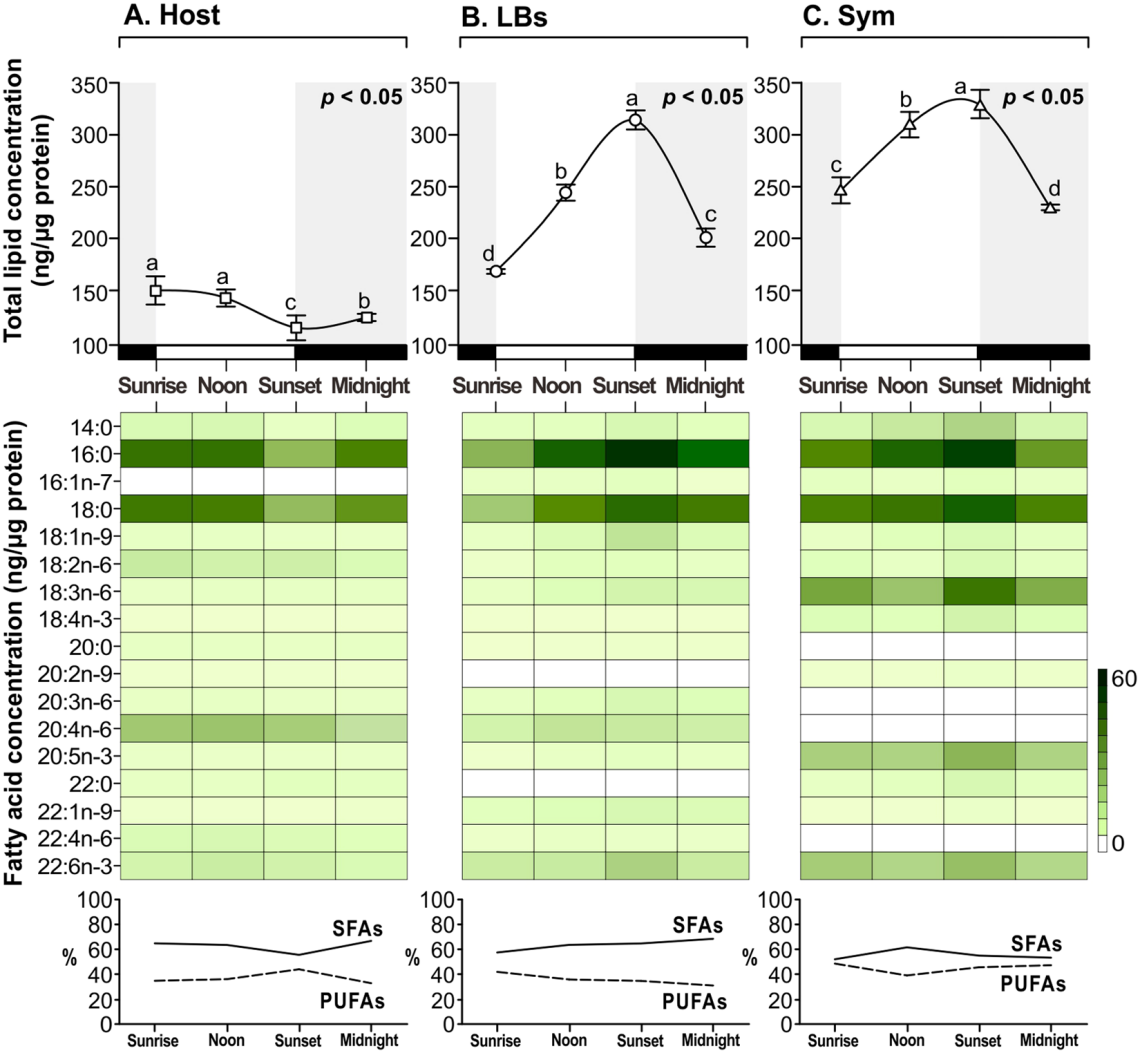
Diel fluctuation of lipid and fatty acid concentrations. The top curve diagram is total lipid concentration over the diel cycle between three cellular compartments of the reef-building coral *Euphyllia glabrescens*: (**A**) host coral gastrodermal cells (“Host,” hollow squares), (**B**) lipid bodies (“LB,” hollow circles), and (**C**) *in hospite Symbiodinium* (“Sym,” hollow triangles). The middle heat map was used to show the changes in concentration of prominent fatty acids (FAs). Bottom is the saturated and polyunsaturated FAs proportion (SFAs and PUFAs). Values (mean ± SD, n = 4) represent the total lipids and FAs concentrations at the respective sampling times, and a significant effect of time on total lipid concentration was documented for all three cellular compartments (one-way ANOVA effect of time, *p* < 0.05 in all cases). Lowercase letters represent significant differences (*p* < 0.05) between individual means, as determined by Tukey’s post-hoc tests. The light/dark period indicates in the open (day time) and closed (night time) boxes.

Dynamic changes in the concentrations of total lipids and corresponding fatty acids (FAs) among host coral gastrodermal cells, LBs, and *in hospite Symbiodinium* were documented over the diel cycle (Fig. 1, Supplementary Tables S1 and S3). The diel fluctuation pattern differed among these three cellular compartments. A general increase in total lipids and FAs concentration was observed in LBs and *in hospite Symbiodinium* during the light period, followed by a decrease during darkness. However, the concentrations of total lipids and FAs in the host gastrodermal cells exhibited the opposite pattern; specifically, concentrations of total lipids gradually declined from sunrise (149.7 ± 13.6 ng/μg protein) to sunset (113.7 ± 12.0 ng/μg protein) and increased at midnight (123.7 ± 3.5 ng/μg protein). This increase during the night was mainly driven by an increase in saturated FAs (SFAs) concentration from sunset (44.8 ± 1.6 ng/μg protein) to midnight (59.5 ± 4.1 ng/μg protein) (Fig. 1A). The lipid saturation proportion in the LBs increased across the day and decreased after midnight (Fig. 1B). Concentrations of SFAs and n–9, n–6, and n–3 polyunsaturated FAs (PUFAs) in the LBs reached peak levels at sunset (94.0 ± 2.5, 13.0 ± 0.2, 21.4 ± 5.1, and 14.0 ± 2.6 ng/μg protein, respectively) and then declined during the night (75.0 ± 1.3, 7.7 ± 1.8, 17.4 ± 0.6, and 8.6 ± 0.6 ng/μg protein, respectively). Finally, SFAs concentration of *in hospite Symbiodinium* increased from sunrise to sunset (Fig. 1C; 61.0 ± 2.0 and 98.7 ± 0.5 ng/μg protein, respectively), whereas PUFAs concentrations increased significantly from noon to sunset (47.7 ± 1.8 and 82.2 ± 4.8 ng/μg protein, respectively).

### Dynamic change in total fatty acid pools

**Figure 2:**
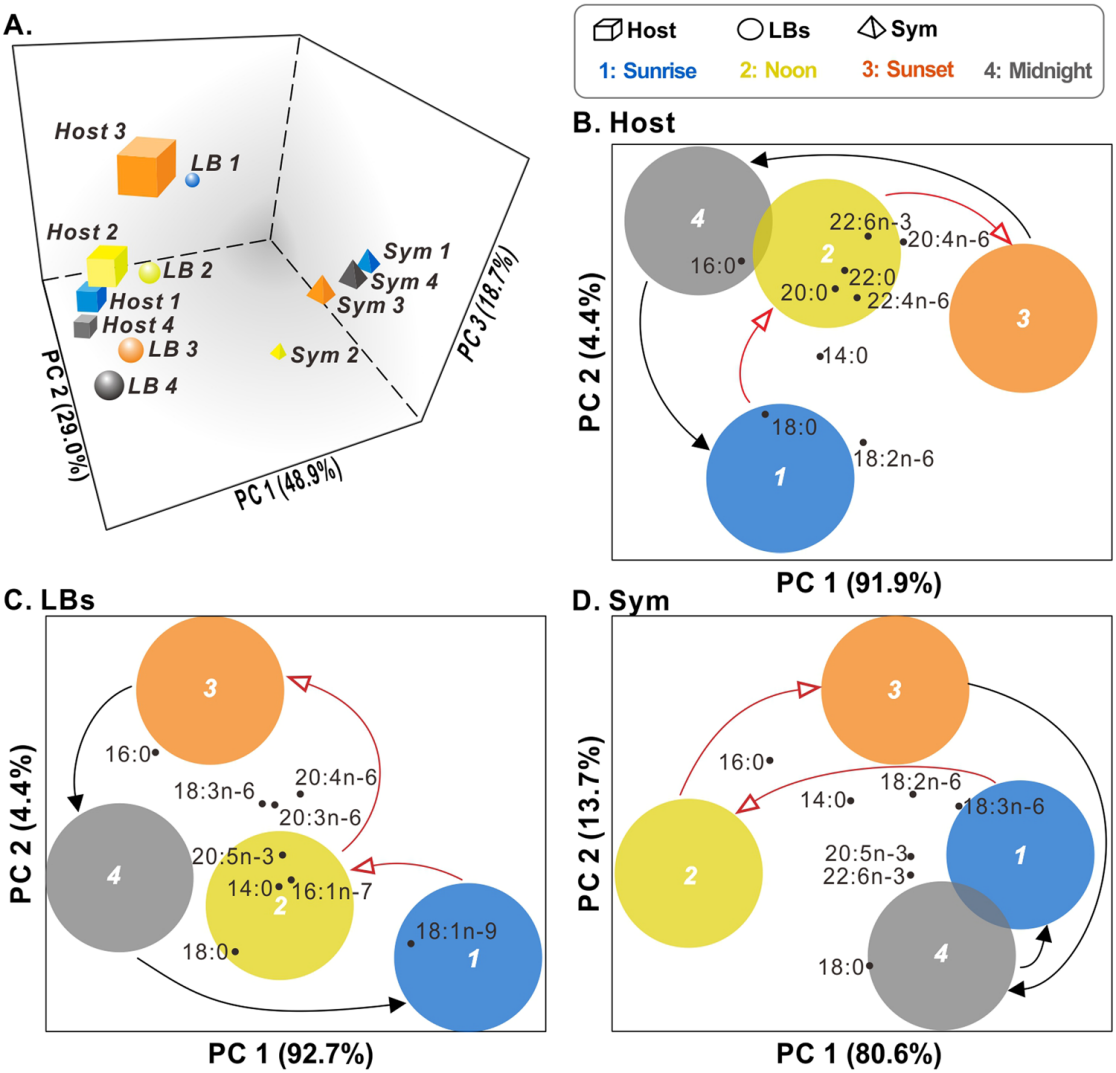
Dynamic change of total fatty acid pools. (**A**) Principal components analysis (PCA) of fatty acid (FA) moieties in the total lipid pools of the three cellular compartments of the *Euphyllia glabrescens-Symbiodinium* endosymbiosis- host coral gastrodermal cells (“Host,” cubes), lipid bodies (“LB,” spheres), and *in hospite Symbiodinium* (“Sym,” tetrahedrons)- across the diel cycle. The PCA bi-plots of individual compartments (**B**) host, (**C**) LB, and (**D**) Sym were included to identify the FAs that contributed most to the variation across the diel cycle. In panels (B), (C) and (D), the red arrows indicate the light period (1: sunrise [blue] and 2: noon [yellow]), and black arrows indicate the dark period (3: sunset [orange] and 4: midnight [gray]).

We used principal components analysis (PCA) and cluster analyses to identify similarities and differences in FA profiles among the three cellular compartments at four sampling time points (Fig. 2, Supplementary Tables S3 and S8). PCA explained 96.6% of the variation, and enabled identification of the prominent FAs that characterized sample clusters. These prominent FAs (those associated with the highest loading scores in the PCA) were found only or primarily in particular sample clusters and contributed most significantly to the principal components (PCs) score. FA moieties varied in concentration over time, and host gastrodermal cells and LBs were more similar to each other than either was to the *in hospite Symbiodinium* over the diel cycle (Fig. 2A). The host fraction FAs (Fig. 2B) with the highest loading score changed over time from C18:0 at sunrise to C20:0, C22:0, C20:4n–6, C22:4n–6, and C22:6n–3 at noon and sunset and then to C16:0 at midnight. The LB fraction FAs (Fig. 2C) with the highest loading score were C18:1n–9 at sunrise, C20:5n–3, C16:1n–7, and C14:0 at noon, and C18:3n–6, C20:3n–6, and C20:4n–6 at sunset. At midnight, C16:0 and C18:0 contributed most to the factor loading score. The lipid species that contributed most to the separation of the *Symbiodinium* group were C18:2n–6, and C18:3n–6 at sunrise, C14:0 and C16:0 at noon, C14:0, C16:0, C18:2n–6, and C18:3n–6 at sunset, and C18:0, C20:5n–3 and C22:6n–3 at midnight (Fig. 2D).

### Variation of individual lipid concentrations

**Figure 3:**
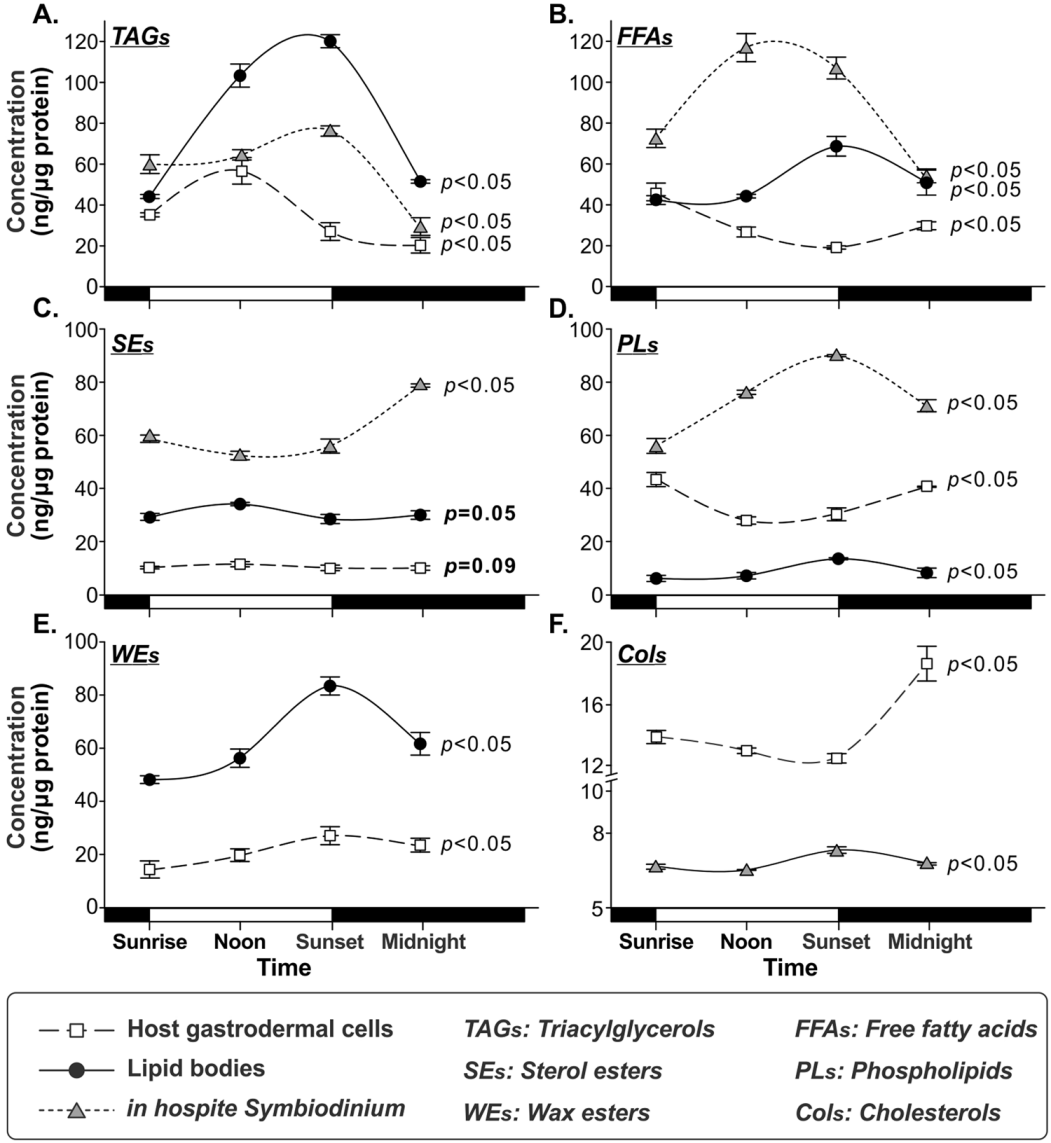
Different variation of individual lipid concentrations. (**A**) TAGs, (**B**) FFAs, (**C**) SEs, (**D**) PLs, (**E**) WEs, and (**F**) Cols (please see main text for full names) among host coral gastrodermal cells (hollow squares), lipid bodies (solid circles), and *in hospite Symbiodinium* populations (gray triangles) over the diel cycle (mean ± SD, n = 4). In the x-axis, the light/dark period indicates in the open (day time) and closed (night time) boxes.

Concentrations of most lipid species varied temporally in all three compartments of the *E. glabrescens–Symbiodinium* endosymbiosis. In the host gastrodermal cells, most lipids were typically found at concentrations between 20 and 60 ng/μg protein (Fig. 3 and Supplementary Table S2). Specifically, TAGs (Fig. 3A) were accumulated from sunrise (35.2 ± 0.2 ng/μg protein) and reached its highest concentration at noon (56 ± 6.8 ng/μg protein); FFAs concentration (Fig. 3B) tended to decrease during the light period (sunrise to sunset, 45.6 ± 2.6 to 19.1 ± 0.4 ng/μg protein). However, SEs (Fig. 3C) were measured at relatively low concentrations (approximately 10 ng/μg protein) and presented a slight peak at noon (*p = *0.09). The concentrations of FFAs, PLs, and Cols in the host coral fraction typically showed a downward trend from sunrise to sunset, followed by an upward trend after sunset (Fig. 3). PLs concentration (Fig. 3D) increased from a low level of 27.9 ± 0.4 ng/μg protein at noon to 40.6 ± 1.9 ng/μg protein at midnight. Finally, WEs (Fig. 3E) continued to increase throughout the course of the day, and reached 27.1 ± 4.1 ng/μg protein at sunset.

The dominant lipid species in LBs were TAGs, FFAs, and WEs (40–100 ng/μg protein each), with concentrations varied significantly over time (Fig. 3 and Supplementary Table S2). The highest concentrations of TAGs (Fig. 3A), FFAs (Fig. 3B), and WEs (Fig. 3E) were measured in samples collected at sunset (120.2 ± 1.6, 83.5 ± 2.6, and 68.7 ± 4.6 ng/μg protein, respectively). Cols were not detected in the LBs. PLs (Fig. 3D) also demonstrated diel variation (sunrise, noon, sunset, midnight; 6.1 ± 1.0, 7.1 ± 0.6, 13.5 ± 1.1, 8.2 ± 1.3 ng/μg protein). SEs concentration (Fig. 3B) increased as light intensity (sunrise to noon, 29.1 ± 1.3 to 34.1 ± 0.3 ng/μg protein).

The main lipid species within the lipidomes of *in hospite Symbiodinium* (Fig. 3 and Supplementary Table S2) were TAGs (Fig. 3A), FFAs (Fig. 3B), SEs (Fig. 3C), and PLs (Fig. 3D; 60–120 ng/μg protein for each species). SEs concentration declined over the course of the day (sunrise to noon, 58.7 ± 0.7 to 52.4 ± 1.1 ng/μg protein) until sunset at which point it began to increase, reaching its highest concentration at midnight (78.7 ± 6.6 ng/μg protein). The most abundant *Symbiodinium* lipids were FFAs, and its concentration reached a peak at noon (117.1 ± 12.5 ng/μg protein). The variation in TAGs and PLs concentrations revealed a similar trend: highest levels at sunset (76.6 ± 1.3 and 90.0 ± 3.3 ng/μg protein, respectively) and lowest at midnight (28.7 ± 6.2 and 71.0 ± 0.1 ng/μg protein, respectively).

WEs (Fig. 3E) were not detected in *in hospite Symbiodinium*. However, four classes of WEs varied in concentration over time in both host gastrodermal cells and LBs (Table 1), reaching peak levels at sunset. The most abundant WE was palmityl palmitate (R = C16/R′ = C16), which reached maximum concentrations at sunset in host gastrodermal cells and LBs (14.5 ± 2.3 and 29.6 ± 1.9 ng/μg protein, respectively). Palmityl oleate (R = C18:1/R′ = C16) was only detectable in LBs, and its concentrations at noon and sunset (the highest value) were 9.9 ± 0.7 and 18.0 ± 1.6 ng/μg protein, respectively.

**Table 1:** Wax esters (WEs) concentrations in host coral gastrodermal cells and lipid bodies (LBs) across the diel cycle.

Compartment	Time	Concentration of wax ester (ng/μg protein)			
		Palmityl myristate (R = C14/R′ = C16)	Palmityl palmitate (R = C16/R′ = C16)	Palmityl stearate (R = C18/R′ = C16)	Palmityl oleate (R = C18:1/R′ = C16)
Host	Sunrise	**2.5 ± **0.5	**8.1** ± 0.5	**3.0 ± **0.5	—
	Noon	**2.5** ± 0.4	**10.4** ± 0.9	**3.4 ± **0.3	—
	Sunset	**3.0** ± 0.6*	**14.5 ± **2.3*	**4.5 ± **0.6*	—
	Midnight	**2.7** ± 0.6	**9.8 ± **0.4	**3.0 ± **0.5	—
	*p*	0.52	<0.05	0.06	—
LBs	Sunrise	**4.8** ± 0.5	**13.8** ± 1.7	**10.8** ± 0.7	**—**
	Noon	**5.9** ± 0.9	**18.4** ± 0.9	**12.0** ± 1.2	**9.9** ± 0.7
	Sunset	**7.5** ± 0.5*	**29.6** ± 1.9*	**15.2** ± 1.1*	**18.0** ± 1.6*
	Midnight	**6.5** ± 0.1	**17.9** ± 1.9	**11.8** ± 1.6	**11.9** ± 1.3
	*p*	<0.05	<0.05	0.06	<0.05
Significant level between two compartments across time *(p)*	<0.005	<0.005	<0.005	<0.005	

Data were analyzed with multi-factor ANOVA to determine the effect of cellular compartment (host vs. LBs) and time for each WEs species (*p < 0.05, **p < 0.005, ***p < 0.001), and letters adjacent to values (concentration ± SD) represent statistically significant differences across sampling times and compartment within a WE species, as determined by Tukey’s *post hoc* tests (*p* < 0.05). The concentrations of all four lipid species were higher in LBs than host gastrodermal cells at all four sampling times (*p* < 0.05; excepting C18:1/R′ = C16 at sunrise), as well as when data were pooled over time for each of the four lipid species (*p* < 0.05). “—” = not detected.

### Temporal contribution of individual lipids to LB formation

**Figure 4:**
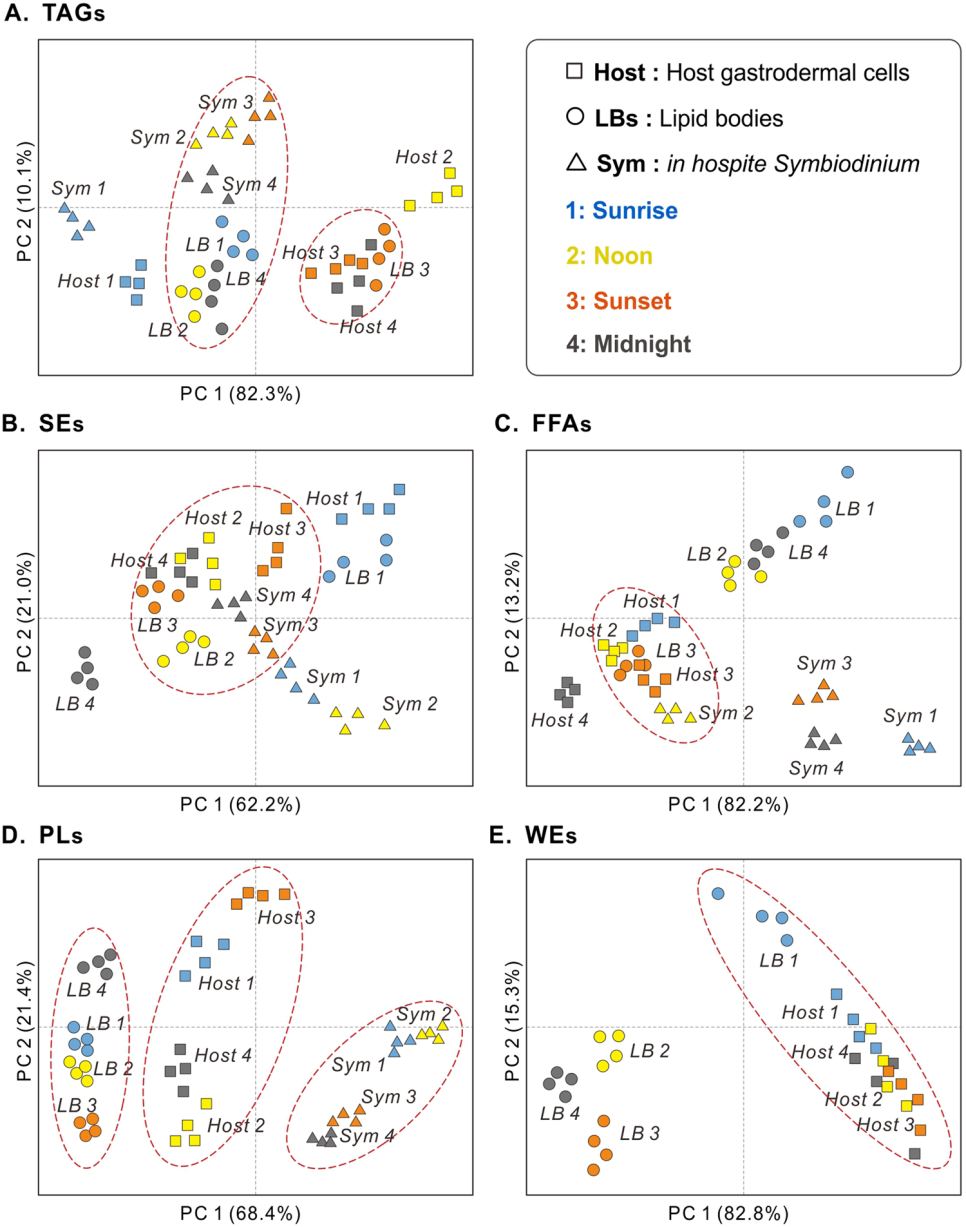
Temporal contribution of individual lipids to LB formation. Principal components analysis of FA moieties in individual lipid classes (**A**) TAGs, (**B**) SEs, (**C**) FFAs, (**D**) PLs, and (**E**) PCA of WE species among host coral gastrodermal cells (“Host,” squares), lipid bodies (“LBs,” circles), and *in hospite Symbiodinium* (“Sym,” triangles) across the diel cycle (blue: sunrise, yellow: noon, orange: sunset, and gray: midnight). The red dash circles presented to cluster the closely relation and strongly similarity of FA moieties after PCA across multiple compartments.

We attempted to understand the LBs formation by investigating its temporal contribution of individual lipids from cellular compartments (i.e., the host cell and symbionts) over the diel cycle. PCA was used to highlight temporal differences in individual lipid species at each sampling time (see Fig. 4, Table 1 and Supplementary Tables S4–S7 and S9). PCA of TAGs (Fig. 4A) revealed an apparent clustering of host at sunset and midnight (Host 3 and 4, respectively) with LBs at sunset (LB 3) along PC1, which explained the vast majority of the variation (>80%). Moreover, there was also a similarity amongst *Symbiodinium* from noon to midnight (Sym 2, 3, and 4) and LBs along PC1. This is partially masked by the fact that these two fractions were also separated along PC2, though this PC account for only 10% of the variation. In the PCA of the FA profile of SEs, the first two PCs explained 62.2% and 21.0% of the variation (Fig. 4B, 83.2% total). In particular, PCA revealed small differences at noon to midnight in the host coral gastrodermal cells (Host 2, 3, and 4), and sunset to midnight in the *in hospite Symbiodinium* populations (Sym 3 and 4, respectively). The PCA suggested that FA moieties of host and *Symbiodinium* SEs were similar to those of LBs during these sampling times. For FFAs (Fig. 4C), the first two PCs explained 95.4% of the total variation (PC1, 82.2%; PC2, 13.2%). Some groups clustered together along PC1, including hosts from sunrise to sunset (Host 1, 2, and 3), LBs at sunset (LB 3), and *in hospite Symbiodinium* at noon (Sym 2). These results indicated that the FA moieties of the host’s FFAs pool resembled those of the LBs from sunrise to sunset, whereas *Symbiodinium* FFAs profiles were similar to those of LBs at noon.

PCA of the PLs dataset (Fig. 4D) showed a clear separation of LBs, host coral, and *in hospite Symbiodinium* samples, with PC1 and PC2 explaining 68.4% and 21.4% of the total variation, respectively. Host coral samples exhibited higher variation among individuals in the score plots than the *Symbiodinium* samples. Moreover, PCA revealed significant differences in PLs FAs composition among host cells, LBs, and dinoflagellates, and no overlaps were detected between host cells and LBs. PCA of the WEs profile showed that sunrise of LBs and all four sampling times of host were clustered (Fig. 4E; PC1, 82.8%; PC2, 15.3%). This result suggested that WEs of LBs had high similarity at sunrise with the host.

### Inter-compartmental lipid involvement

**Figure 5:**
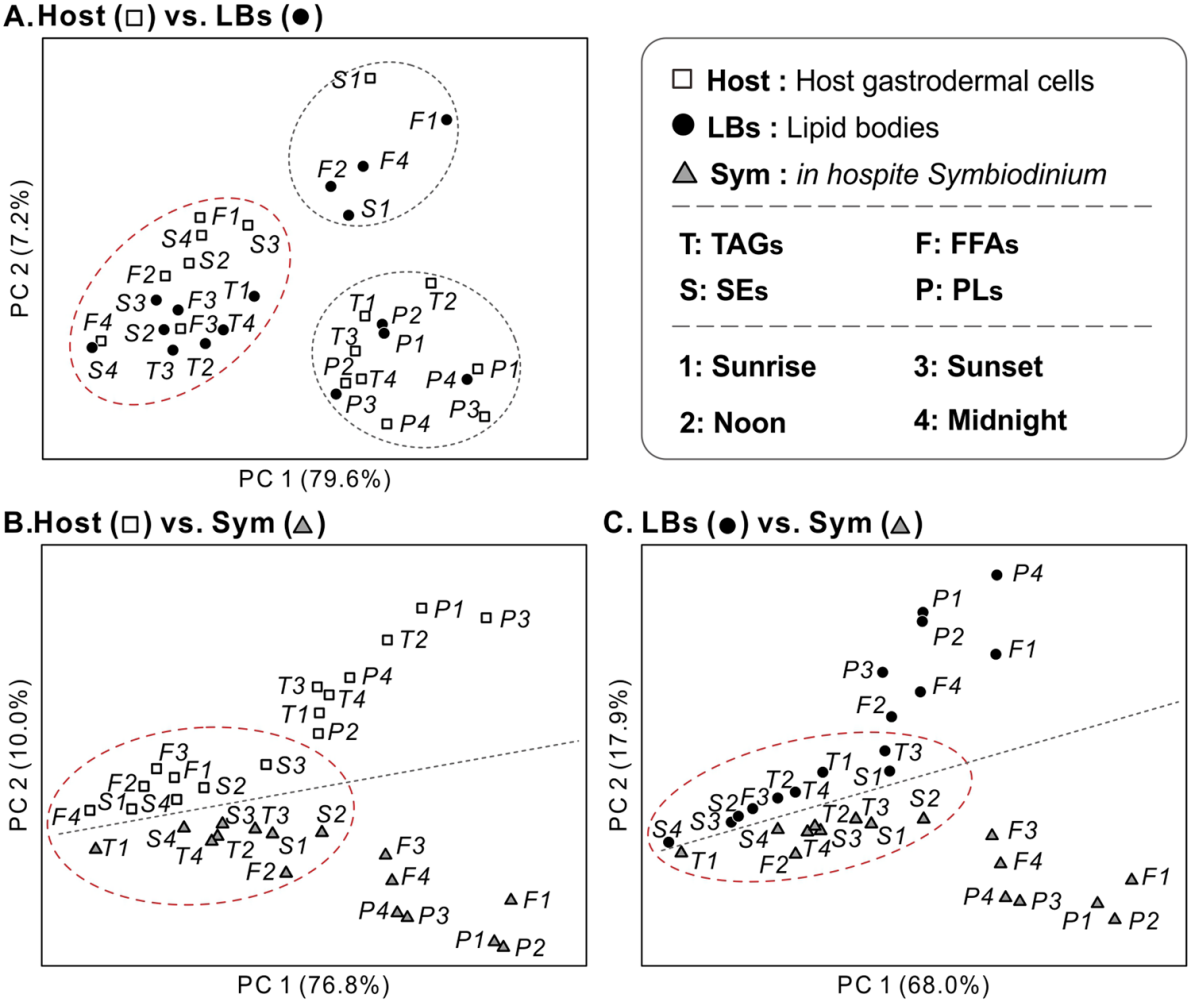
Inter-compartmental lipid involvement. Principal components analysis (PCA) of fatty acid (FA) moieties in individual lipid species within and across multiple compartments of the *Euphyllia glabrescens-Symbiodinium* endosymbiosis. (**A**) host gastrodermal cells (“Host,” hollow squares) versus lipid bodies (“LBs,” solid circles) (**B**) host (hollow squares) versus *in hospite Symbiodinium* (“Sym,” gray triangles) (**C**) LBs (solid circles) versus Sym (gray triangles) across the diel cycle (1: sunrise, 2: noon, 3: sunset, and 4: midnight). T: TAGs, F: FFAs, P: PLs, and S: SEs (see main text for full names of these lipids). The red dash circles presented to cluster the closely related of lipid species and strongly similarity of FA moieties after pairwise comparison of PCA across multiple compartments.

PCA was used to visualize variation in seventeen FA moieties of different lipids across the three compartments over the diel cycle (Fig. 5 and Supplementary Tables S4–S7 and S10). First, three major groups of lipids were shared and overlapped to some degree between host gastrodermal cells and LBs (Fig. 5A). Particularly, TAGs and SEs of LBs had FAs compositions similar to those of FFAs and SEs in host gastrodermal cells. Moreover, there were minor differences in FAs compositions between the PLs of LBs and PLs and TAGs of host gastrodermal cells. As shown in Fig. 5B, host gastrodermal cells and *in hospite Symbiodinium* were sorted into two groups (host and *Symbiodinium* data are above and below the dotted diagonal line, respectively). Only the FFAs and SEs of the host and TAGs and SEs of the *in hospite Symbiodinium* comprising PC2 were clustered. Finally, LBs and *Symbiodinium* were also well separated through PCA (Fig. 5C), and the lipid profiles of these two compartments varied at all sampling times. They were relatively similar only with respect to TAGs, SEs, *Symbiodinium* F2 (FFAs at noon), and LB F3 (FFAs at sunset).

Further PCA on individual cellular compartments (i.e., the host gastrodermal cell, LBs and symbionts; Supplementary Figs S1–S3 and Supplementary Table 11) to evaluate the distribution of prominent FAs demonstrated that several *Symbiodinium*-derived FAs, including C18:2n–6, C18:3n–6, C18:4n–3, C20:5n–3, and C22:6n–3 (Supplementary Fig. S3) were also characteristic in the SEs, TAGs and FFAs clusters of both the host gastrodermal cells (Supplementary Fig. S1) and LBs (Supplementary Fig. S2). On the other hand, the prominent FAs of host gastrodermal cells, including C20:3n–6, C20:4n–6, and C22:4n–6, are also characteristic in SEs, TAGs and FFAs of LBs (Supplementary Figs S1 and S2).

## Discussion

Coral lipids profiles and FAs composition are related to the mode of nutrient acquisition, and lipidomes may be strongly affected by symbiotic status and environmental conditions^7, 12, 21, 22^. Understanding the dynamic change of the lipidome enables the elucidation of endosymbiosis regulation. However, lipid profiling in anthozoan–dinoflagellate mutualisms is complicated by the dual-compartmental nature of the holobiont, an entity whose molecular regulation remains unclear^23^. Furthermore, temporal changes in LB distribution, composition, and morphology, all of which appear to suggest that the host-*Symbiodinium* endosymbiosis is quite dynamic over the diel cycle with respect to metabolite production and catabolism^14^. In the present research, we investigated the nature of endosymbiosis through lipidomic analysis using a time-varied compartmental approach, and revealed the changes in marker lipids and LBs as regulators during metabolism pathways.

### Temporal dynamic of endosymbiosis status

In this study, we sought to unravel a portion of the lipidome molecular regulation of an anthozoan–dinoflagellate endosymbiosis in three cellular fractions: the host gastrodermal cells, LBs, and *in hospite Symbiodinium* populations, at four points of the diel cycle. Total lipids and FAs pools exhibited diel variation in all three cellular compartments; particularly, the overall lipid profile of the host showed a distinct diel fluctuation pattern compared with that of the other two compartments (Fig. 1). Essentially, the diel variation in holobiont lipidome (discussed in detail in further sections) is likely to have critical implications in the maintenance of symbiotic homeostasis^24, 25^.

In particular, there is a relatively diel pattern of FA moiety abundance which differs between host cells and LBs (Fig. 1). SFAs and PUFAs are essential for biological membrane synthesis and play a critical role in the regulation of coral metabolism^26^. The proportion of SFAs and PUFAs revealed a distinct temporal pattern, indicating that the lipogenesis in individual compartments of the holobiont varies over the diel cycle. As shown in Fig. 1C, *Symbiodinium* FA moieties accumulated during the day and were consumed at night, exhibiting a circadian lipogenesis pattern. Acetyl-CoA carboxylase is a critical light-dependent enzyme that regulates the FA synthesis rates^27, 28^. Light-driven lipogenesis by microalgae, which has been thoroughly studied by Crossland and Mortillaro *et al*., may have contributed to the temporal variation in relative FA levels^29, 30^. Previous studies have shown that marine invertebrates and algae typically synthesize more SFAs during the day and more PUFAs at night^31, 32^. This finding may indicate that acyl chains of host cells and *Symbiodinium*, which are necessary for the construction of more complex lipid species, are involved in LB lipogenesis during the daylight hours, accounting for the decrease in host coral SFAs during this period. Thus, the lipid metabolites of the holobiont are accumulated into LBs and influenced by the diel cycle and internal cellular metabolic balance. This hypothesis suggests that lipid trafficking and lipogenesis associated with LB formation causes temporal variation of lipid metabolism in animal cells.

Previous studies have determined that many LBs were typically only found within host cells housing endosymbionts, but not in non-symbiotic host cells; this finding highlights the importance of these organelles in endosymbiosis^12, 14^. Specifically, LB density and lipid concentrations decreased to baseline levels during the night, which may imply that corals are highly susceptible and less resistant to environmental stress. In the present study, we characterized the diel fluctuations of lipid profiles in healthy coral LBs under normal illumination (Figs 1–3). We observed that the FAs pools in each compartment varied from sunrise to midnight (Fig. 2), and identified the prominent FAs that contributed most to the variation in the PCA plot (Fig. 2B–D). Our results of a dynamic lipidome in the animal coral–plant algae interaction clearly suggest that coral–*Symbiodinium* endosymbiosis is a temporally variable status. In addition to shedding light on the underlying biology of this environmentally crucial endosymbiosis, previous studies^33, 34^ have evaluated the suitability of various approaches for estimating coral health based on a variety of parameters. However, no study has investigated the diel changes in lipid metabolic indices (e.g., lipids, FAs, SFAs, and PUFAs) among various cellular compartments simultaneously. Our results provide valuable lipidomic indices for evaluating coral endosymbiotic status at various timescales and light levels.

### Temporal lipogenesis changes result in diel rhythmicity of LB formation

**Figure 6:**
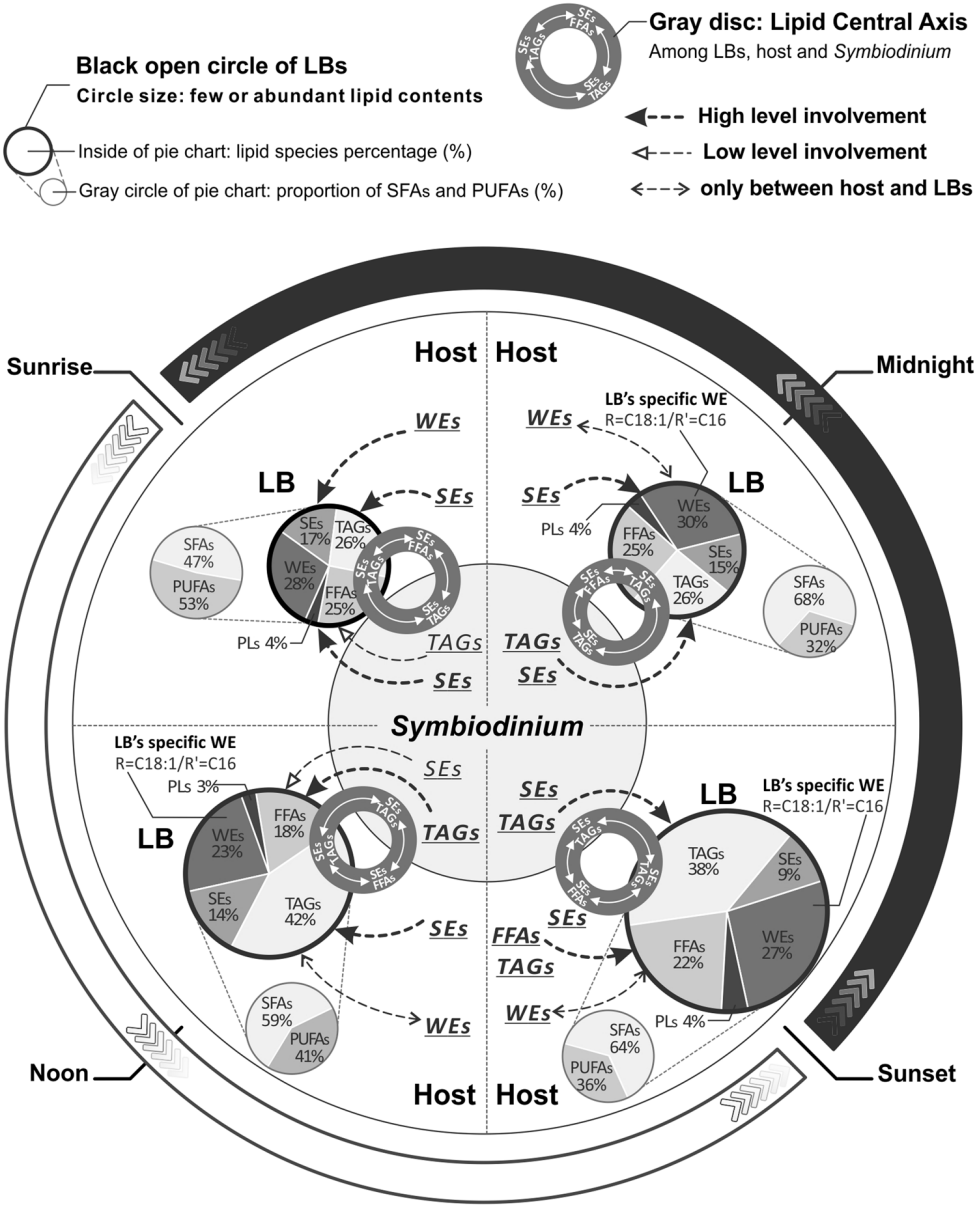
Schematic summary of the temporal LB lipogenesis in coral-*Symbiodinium* endosymbiosis during diel cycle. The circle size of bold black open circles indicative of the total lipid abundance of LBs, and the contained pie charts show percentage of individual lipid species. Furthermore, the extending gray dashed line and gray circle of pie chart presented the proportion of saturated and polyunsaturated FAs in total lipids of LBs. The gray disc among LBs, host and *Symbiodinium* shows the inter-compartmental lipid involvement of lipid central axis. The filled and blank arrows indicate lipids likely to be involved in LB biogenesis with high and low level, respectively. The double-headed arrow presented lipids were exchanged between host and LBs. [1]–[4] indicated four time periods.

The diel variability in LB formation observed by Chen *et al*. is closely related to the dynamic changes in lipid composition observed in the present study^14^. Many lipidomic studies have revealed that TAGs and SEs serve as intracellular storage molecules accumulated in LBs^35, 36^. The coral hosts accumulated these storage lipids during the light period (Fig. 3A and C) and synthesized the structural lipids PLs and Cols at night (Fig. 3D and F), as previously reported^37^. Nevertheless, the temporal contribution of individual lipids pools to LB formation revealed that *Symbiodinium* derived TAGs were involved in the lipogenesis of LBs after sunrise, while the involvement of host derived TAGs was highest at sunset (Figs 4A and 6, [3]). Furthermore, SEs are essential storage molecules of cellular membrane components and are synthesized by cholesterol acyltransferase within the endoplasmic reticulum, or through an acyl-CoA-independent reaction by lecithin^35, 38^. Circadian changes in SEs metabolism have been related to sterol esterase activity, which appears to be activated by sterols and acyl donors^39, 40^. In the present study, FFAs and PLs concentrations of host cells showed a diel fluctuation trend antagonistic to that of SEs in LBs and host cells (Fig. 3). Also, it is highly implied that host SEs play a role in lipid metabolism of LBs throughout the day (Figs 4B and 6). This suggests that host cells may use FFAs and PLs as acyl donors to synthesize more SEs through LB formation during the day. Moreover, SEs synthesizing by *Symbiodinium* are likely, and involved in LB formation in the afternoon (Figs 3C, 4B and 6).

Our finding that TAGs and FAs synthesis occurred primarily during the light period followed by a rapid decrease in the dark is in agreement with other studies which demonstrated that some key enzymes of the TAGs and FAs synthesis pathways are regulated by light and are involved in lipid-droplet or LB biosynthesis^31, 41^. Furthermore, FA moieties of the host FFAs showed a strong similarity with those of LBs at sunset (Fig. 4C). This may indicated that the host contributes FFAs to LB biogenesis at this time (Fig. 6, [3]). This may be attributed to the importance of FFAs as building blocks of other lipids, and likely suggests a rapid lipid turnover and degradation in host cells, which mobilized implicated lipid metabolites (TAGs and SEs) from *Symbiodinium* and involved in LB formation. Apart from this, PLs, which are typically structural components in membranes, exhibited significant differences among the three compartments across the diel cycle (Fig. 4D). As shown in other species, LBs originate from the endoplasmic reticulum in other eukaryotic cells, and the FAs composition of PLs varies among organelles^42, 43^.

WEs of LBs, specifically saturated WEs, reached the highest concentrations at sunset (Table 1), coinciding with the highest density of electron-transparent inclusion bodies (ETIs) visualized in Chen *et al*.^14^. It had been hypothesized that the predominant lipid species of these ETIs are WEs. WEs are not detected in *Symbiodinium* and are only present in host cells and LBs^16, 44^. Previous studies have demonstrated that *Symbiodinium* may provide unesterified FAs, which are then reduced to fatty alcohol in the host and esterified with FAs to form WEs during the day^41, 45^. Our data clearly indicate that coral animal cells contain substantial and dynamic quantities of WEs across the diel cycle. Nevertheless, LB migration across the gastroderm and the consequent density changes might imply that coral host cells utilize and metabolize the lipid pools of the LBs at night^14^. Moreover, host WEs may be used to generate LBs at sunrise (Figs 4E and 6, [1]). These findings mean that lipids synthesized in the host cells by endoplasmic reticulum mediated lipogenesis are accumulated within LBs through the lipogenesis mediated by coral host during the light period^13, 14, 43^. Collectively, our data and previous studies suggest that the diel change in the lipid composition of LBs, in particular, may be intricately linked to the diel rhythm in their cellular abundance documented in previous studies.

### LBs as a lipid exchange point for lipid metabolites in endosymbiosis

We observed that each compartment possesses a unique lipidome (Fig. 1); furthermore, PCA was used to gain insight into the diel variability in the three lipidomes of the *E. glabrescent*–*Symbiodinium* holobiont, and several FAs were especially important in explaining variation (Supplementary Figs S1, S2, S3 and Table S11). Some of these prominent FAs were only synthesized by *Symbiodinium*, including C18:2n–6, C18:3n–6, and C18:4n–3. as verified in previous studies^10, 16, 46, 47^. Moreover, C20:3n–6, C20:4n–6, and C22:4n–6 were produced by the host only, which is also supported by previous studies^16, 48^. This demonstrates that TAGs, SEs, and FFAs are among the most trafficked lipid species among different cellular compartments. Moreover, such trafficking, as the lipid central axis, may occur through the LBs (Figs 5 and 6). Nevertheless, some FAs were only detectable in specific lipid species of either host cells and LBs, host cells and *Symbiodinium*, or only in LBs and *Symbiodinium*. Possible trafficked lipids and FA moieties from *Symbiodinium* to the host^10, 44^, or host to *Symbiodinium* ^49^ have been studied in hard corals for decades. However, the direct translocation of lipids and lipogenesis among LBs, coral host and *Symbiodinium* has not been reported^9, 12, 47^.

It has been hypothesized that *Symbiodinium* synthesize sugars, lipids, and carbohydrates, which are then transported into the host (and LBs) during endosymbiosis^7, 45^. In this study, we presented a hypothetical model of schematic summary of the temporal LB lipogenesis (Fig. 6) during a diel cycle. First, LB formation is endosymbiosis-dependent. According to the present LB lipidome, a previous conclusion based on LB morphological and distribution changes was further confirmed^12, 14^: the endosymbiosis is dynamic rather static, and shows a diel pattern. For example, the marker organelles of endosymbiotic status, LBs, in total lipid concentration, lipid species composition, and FA moieties change dynamically from sunrise to midnight (Fig. 6, [1]–[4]). Second, we hypothesize that there are also temporal changes of lipid flow within holobiont lipogenesis, according to diel examination which leads to two major discoveries: (a) Lipid transport occurred in both host and symbiont, and LB was the relay center across the day (Fig. 6, [1]–[4]). Inter-compartmental lipids transport featured predominantly TAGs, SEs, and FFAs, which as the lipid central axis among the LBs, host, and *Symbiodinium*. Furthermore, WEs were only present in the host cells and LBs, suggesting that WEs trafficking only occurred between these compartments. (b) LB formation, particularly, the lipid compositions were temporally regulated by the activation of various pathways. For example, TAGs of *Symbiodinium* were involved in LB lipogenesis from noon to midnight (Fig. 6, [2]–[4]); the host were highly contributed TAGs to LB lipogenesis at sunset (Fig. 6, [3]); SEs of *Symbiodinium* may have participated in LB biogenesis from sunset to midnight and subsequently again at sunrise (Fig. 6, [3]–[4] followed by [1]); SEs and FFAs of the host were associated with LB formation during the light period (Fig. 6, [1]–[3]); WEs of the host were initially implicated in the biogenesis of LB at sunrise (Figs 6, [1]). Yet, the WEs species, palmityl oleate, was only present in LBs from noon to midnight (Fig. 6, [2]–[4]).

In our study, compartment-specific and temporal changes in the lipidome of a hermatypic coral were analyzed, which provided experimental evidence of the temporally variable status of coral–*Symbiodinium* endosymbiosis. The diurnal rhythmicity of LB formation documented in prior studies may have driven by such the temporal lipidomes of dynamic lipogenesis. Furthermore, our data show that LBs serve as a critical relay center for lipid metabolites trafficking between host and endosymbionts, meaning that they are key regulators of this ecologically important endosymbiosis. According to our review of relevant literature, the present investigation is the first to explore diel fluctuations in the lipidomes of all three compartments in the endosymbiotic coral holobiont. Future studies must investigate the role of lipids trafficking and monitor the lipidomics of host gastrodermal cells under various photosynthetic states.

## Materials and Methods

### Coral collection and maintenance

Sixteen colonies of the reef-building coral *Euphyllia glabrescens* (7.2 ± 1.2 to 35.8 ± 2.3 cm in diameter) were collected from fringing reefs abutting the inlet of a power plant in Southern Taiwan (21°57′22.6″N, 120°45′17.5″E). The coral collection was approved by the Kenting National Park Management Office in Kenting, Taiwan. Colonies were transported within 1 h to the husbandry center of the National Museum of Marine Biology and Aquarium (NMMBA) and placed in individual outdoor tanks (4 kL) with flow-through seawater (exchange rate = 80 L/hr). The temperature was maintained at 26.5 ± 2 °C using a cooler (First FC-45, Aquatech, Kaohsiung, Taiwan) under a natural photoperiod (12 L:12D). This temperature is similar to the annual mean at the site of collection^50^.

On average, each of the 16 colonies within our culture tank possessed 5–10 polyps and a total of at least 500 tentacles. For one 24-hr diel cycle we sampled a total of 80 tentacles for each of the four time points: sunrise, noon, sunset, and midnight. Tentacles were amputated from polyps of *E. glabrescens* colonies using curved surgical scissors (stretched length of tentacle is approximately 3 cm). Experimental sampling was performed on the 16 colonies were randomly split into two batches of eight colonies. The first batch was sampled at sunrise and sunset, and the second batch was sampled at noon and midnight. For a given sampling time, ten tentacles were removed from each of the eight colonies and pooled to give the total of 80. Additionally, we removed tentacles from various areas around each colony and avoided sampling a single polyp twice within a given sampling day. Thereby, 20 tentacles were taken from a single colony on each sampling day which represents <5% of the whole colony. The experiment was repeated on four different dates (n = 4) with several weeks or months between sampling days. The precise sampling time points on each of these four days was dependent on the light intensity of the photoperiod, i.e. on the approximate time of sunrise (0530–0630), noon (1130–1230), sunset (1730–1830), and midnight (2330–0030). Light intensity and temperature were logged using HOBO^®^ Pendant (Onset, Pocasset, MA, USA) meters placed near the corals in each of the tanks. A detailed treatise of the light environment in our partially shaded, indoor aquarium facility can be found in a prior work^51^. The tentacles were transferred to the laboratory and washed with filtered seawater (FSW) for further analysis.

### Lipid analysis of cellular compartments

The purity determination of the separated fractions (i.e., host gastrodermal cell lysates, LBs, and *in hospite Symbiodinium*) were implemented according to methods by Peng *et al*. and Chen *et al*.^13, 14^. Lipids were extracted by following the Bligh and Dyer^52^ procedure, and the lipid extracts were analyzed through high-performance liquid chromatography (HPLC; Hitachi, Tokyo, Japan). Each lipid class was quantified and fractionated by injecting samples using an auto-injector (Hitachi, L7200) into a normal phase column (YMC-PVA-SIL, 120 nm, 150 × 3 mm ID, 5 μm particle size; YMC America, Allentown, PA, USA). The column was placed in an oven (Hitachi, L7300) at 50 °C during analysis. Gradient programs were developed for the separation of lipids, and a gradient elution scheme according to Chen *et al*. was followed^16^. The elution gradient was delivered using pumps from Hitachi (L7100), and a volume of 30 μL was injected. The lipids were detected using a Sedex 80 evaporative light scattering detection unit (Sedere, France) maintained at 70 °C with a flow of compressed nitrogen gas set at 2.5 kg/cm^2^. The sensitivity of the photomultiplier was adjusted to a gain of five, and the detector signal was recorded and integrated using the Hitachi D-7000 HPLC System Manager software. The solvent system individually fractionated eight major lipid class standards by retention time (as described by Chen *et al*.^16^). The concentration of each lipid class was calculated on the basis of the equations from their integrated areas by solving for the standard calibration curves.

### Fatty acid and wax ester content determination

Fatty acids (FAs) were converted to their FA methyl esters (FAMEs) through transmethylation of the extracted lipids. Specifically, FAMEs were obtained through refluxing in 5 mL of a reagent containing concentrated sulphuric acid–toluene–methanol (1:10:20 v/v/v) and an internal standard (nervonic acid, Sigma-Aldrich) for 2 hr at 90 °C. Subsequently, 5 mL water containing 5% sodium chloride was added, and the esters were extracted with 5 mL hexane using Pasteur pipettes to separate the layers. Subsequently, the hexane layer was washed with 4 mL water containing 2% potassium bicarbonate and dried over anhydrous sodium sulfate. The solution was filtered, and the FAMEs were then ready for injection.

Analysis of FAMEs was performed using a GC-Varian 320 MS CP-3800 System (Varian, Agilent, Austin, TX, USA) equipped with a CP-Sil88 (20 m × 0.25 mm ID, 0.2 μm film thickness, Agilent) capillary column operated in full scan mode (scan range from 100 to 1000 m/z). A sample volume of 1 μL was injected, and helium was used as the carrier gas (flow rate, 1 mL/min). The temperature regime was 50 °C for 1 min, 50–200 °C at 8 °C/min for 5 min, and 200–230 °C at 20 °C/min. Gas chromatography followed by mass spectrometry (GC–MS) was also used for wax esters (WEs) identification. The GC–MS analysis of WE required a capillary column of 30 m in length and 0.25 mm in diameter, and the stationary phase had a film thickness of 0.25 μm (Agilent VF-5ms; Agilent). The temperature program was 40 °C for 1 min, 40–300 °C at 25 °C/min for 15 min, and 300–320 °C at 2 °C/min for 5 min. Spectra were compared with the National Institute of Standards and Technology library (NIST02, Gaithersburg, MD, USA) and the FA mass spectra archive using Saturn GC/MS Workstation software (ver. 6.9.3; Varian). The FAMEs and WEs peaks were analyzed by comparing their retention time with those of the standards as described by Chen *et al*.^16^. The concentrations of individual FAs and WEs species were calculated from the integrated area and normalized to total protein (μg) as described by Chen *et al*.^16^.

### Statistical analysis

Data were expressed as mean ± SD (standard deviation), and Kruskal–Wallis tests were typically used as an alternative to parametric analysis of variance (ANOVA) to determine temporal differences given the tendency of the datasets to lack normality (Shapiro–Wilk’s test *p* < 0.05). Mann–Whitney post hoc U tests were used to determine individual mean differences when a significant difference was detected in the overall ANOVA model (*p* < 0.05). Differences in lipid species among the three cellular compartments across the four time points were analyzed using repeated-measures ANOVA followed by Tukey’s multiple-range procedure (*p* < 0.05).

Principal component analysis (PCA) was used to identify meaningful patterns and associations of individual FAs data. First, the PCA of each cellular compartment was conducted to evaluate the distribution of FAs species contributing the highest loading scores to the PCA plot (Prominent FAs: loading factor on components indicated significant contributions). Second, FA moieties in individual lipid species among the three cellular compartments across the diel cycle were compared using PCA. Finally, the lipid metabolite variability and differences in multiple comparisons of FA moieties in individual lipid species were assessed within each cellular compartment, across the dataset as a whole, and across the following specific comparisons: host vs. *Symbiodinium*, host vs. LBs, and LBs vs. *Symbiodinium*. Furthermore, we labeled the sampling time as follows: 1: sunrise, 2: noon, 3: sunset and 4: midnight, to represent individual time-point in all PCA diagrams. Statistical analyses for all datasets were performed using the Statistical Package for the Social Sciences (SPSS ver. 17.0, IBM, Armonk, NY, USA).

## Electronic supplementary material

Supplementary Information
