# Supplementary material for: Coral lipid bodies as the relay center interconnecting diel-dependent lipidomic changes in different cellular compartments
Source: Sci Rep. 2017 Jun 12;7:3244. doi: 10.1038/s41598-017-02722-z (PMC5468245; doi:10.1038/s41598-017-02722-z)
Supplement: Supplementary file 1 — Supplementary Information [file 41598_2017_2722_MOESM1_ESM.pdf]

Supplementary Information

Coral lipid bodies as the relay center interconnecting diel-dependent lipidomic changes in different cellular compartments

Hung-Kai Chen[1], Li-Hsueh Wang[2,3], Wan-Nan U. Chen[4], Anderson B. Mayfield[3,5], Oren Levy[6], Chan-Shing Lin[1*] & Chii-Shiarng Chen[1,2,3*]

[1]Department of Marine Biotechnology and Resources, National Sun Yat-sen University, Kaohsiung 804, Taiwan

[2]Graduate Institute of Marine Biology, National Dong-Hwa University, Checheng, Pingtung 944, Taiwan

[3]Taiwan Coral Research Center, National Museum of Marine Biology and Aquarium, Checheng, Pingtung 944, Taiwan

[4]Department of Biological Science and Technology, I-Shou University, Kaohsiung 824, Taiwan

[5] Khaled bin Sultan Living Oceans Foundation, Annapolis, MD 21403, United States of America

[6]The Mina and Everard Goodman Faculty of Life Sciences, Bar Ilan University, Ramat Gan 52900, Israel

*These authors contributed equally to this work. Correspondence and requests for materials should be addressed to CSC (email: cchen@nmmba.gov.tw).

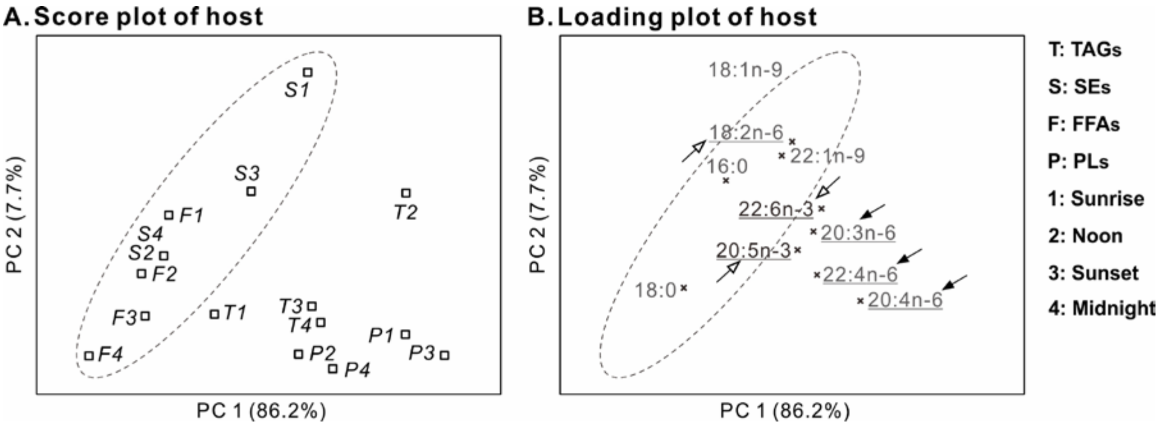

Figure S1. Principal components analysis (PCA) score and loading plot of fatty acids (FA) in individual lipid species of coral host gastrodermal cells. (A) Score plot of host gastrodermal cells ("Host," hollow squares) across the diel cycle (1: sunrise, 2: noon, 3: sunset, and 4: midnight). T: TAGs, F: FFAs, P: PLs, and S: SEs (see main text for full names of these lipids). (B) The PCA loading plot of host was included for prominent FAs. Dark gray exes (×) with solid arrows indicate the prominent FAs from animal host, and hollow arrows highlight the prominent FAs from algae *Symbiodinium* in individual cellular compartments. Dashed-line circles presented to cluster in individual cellular compartments indicated to relate with lipids from light gray circles after PCA pairwise comparison (Fig. 5).

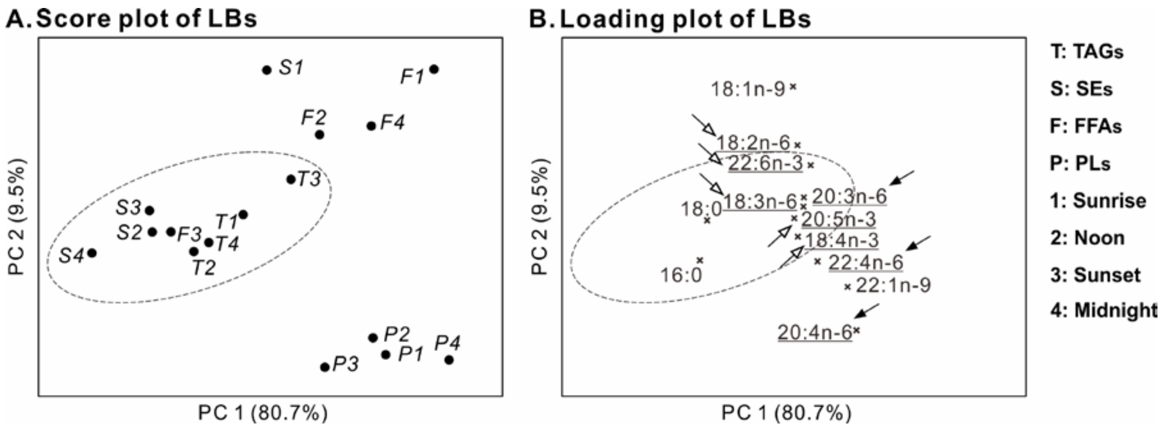

Figure S2. Principal components analysis (PCA) score and loading plot of fatty acids (FA) in individual lipid species of LBs. (A) Score plot of lipid bodies ("LBs", solid circles) across the diel cycle (1: sunrise, 2: noon, 3: sunset, and 4: midnight). T: TAGs, F: FFAs, P: PLs, and S: SEs (see main text for full names of these lipids). (B) The PCA loading plot of LB was included for prominent FAs. Dark gray exes (×) with solid arrows indicate the prominent FAs from animal host, and hollow arrows highlight the prominent FAs from algae *Symbiodinium* in individual cellular compartments. Dashed-line circles presented to cluster in individual cellular compartments indicated to relate with lipids from light gray circles after PCA pairwise comparison (Fig. 5).

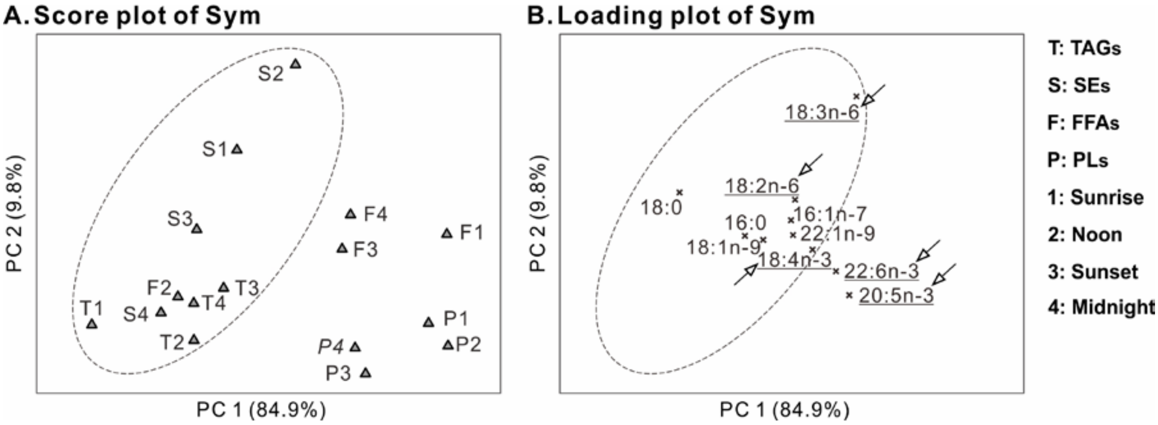

Figure S3. Principal components analysis (PCA) score and loading plot of fatty acids (FA) in individual lipid species of *in hospite Symbiodinium*. (A) Score plot of *in hospite Symbiodinium* ("Sym," gray triangles solid circles) across the diel cycle (1: sunrise, 2: noon, 3: sunset, and 4: midnight). T: TAGs, F: FFAs, P: PLs, and S: SEs (see main text for full names of these lipids). (B) The PCA loading plot Sym was included for prominent FAs. Dark gray exes (×) with hollow arrows highlight the prominent FAs from algae *Symbiodinium* in individual cellular compartments. Dashed-line circles presented to cluster in individual cellular compartments indicated to relate with lipids from light gray circles after PCA pairwise comparison (Fig. 5).

Table S1. Concentrations of total lipid saturated FAs (SFAs)/ polyunsaturated FAs (PUFAs) saturation ratios from host coral gastrodermal cells (Host), lipid bodies (LBs), and *in hospite Symbiodinium* (Sym) across the diel cycle. Concentrations of total lipid dataset were analyzed with ANOVA followed by Duncan's multiple-range procedures to determine the effect of different sampling times (*$p<0.05$, ** $p<0.005$, *** $p<0.001$). Letters adjacent to values (concentration±SD) represent statistically significant differences across sampling times. "—" = not detected.

			%	
Cellular compartments	Time	Total lipid concentration (ng/µg protein)	SFAs	PUFAs
Host	Sunrise	149.7±13.6[a]	65.0	35.0
	Noon	142.4±8.3[a]	63.7	36.3
	Sunset	113.7±12.0[c]	55.8	44.2
	Midnight	123.7±3.5[b]	66.9	33.1
	p	**	—	—
LBs	Sunrise	170.0±2.3[d]	57.8	42.2
	Noon	245.1±7.7[b]	63.8	36.2
	Sunset	314.3±9.1[a]	64.9	35.1
	Midnight	202.2±8.7[c]	68.7	31.3
	p	***	—	—
Sym	Sunrise	247.3±12.4[c]	51.7	48.3
	Noon	309.9±12.1[b]	61.1	38.9
	Sunset	329.5±13.5[a]	54.6	45.4
	Midnight	231.1±2.7[d]	53.0	47.0
	p	***	—	—

Table S2. Concentrations of individual lipid species (see main text for full name of each species.) from host coral gastrodermal cells (Host), lipid bodies (LBs), and *in hospite Symbiodinium* (Sym) across the diel cycle. Data were analyzed with ANOVA followed by Duncan's multiple-range procedures to determine the effect of cellular compartment for each of 17 fatty acids (*p<0.05, ** p<0.005, *** p<0.001), and Kruskal-Wallis tests were used to determine the effect of sampling time. Letters adjacent to values (concentration±SD) represent statistically significant differences across sampling times and compartment within a lipid species, as determined by Mann-Whitney post-hoc U tests (p<0.05). "—" = not detected.

		Concentration of lipid species (ng/µg protein)					
Time		**WEs**	**SEs**	**TAGs**	**Cols**	**FFAs**	**PLs**
	**Host**	14.2±3.5^{b}	10.2±0.2^{c}	35.2±0.2^{c}	13.9±0.5^{a}	45.6±2.6^{b}	43.6±1.4^{b}
	**LBs**	48.1±1.1^{a}	29.1±1.3^{b}	44.2±0.5^{b}	—	42.5±1.1^{b}	6.1±1.0^{c}
Sunrise	**Sym**	—	58.7±0.7^{a}	60.0±2.8^{a}	6.7±0.1^{b}	72.5±5.6^{a}	56.0±3.4^{a}
	F	821	4967	340	3526	127	421
	p	***	***	***	***	***	***
	**Host**	19.6±3.2^{b}	11.4±0.6^{c}	56.9±6.8^{b}	13.0±0.1^{a}	26.7±1.2^{c}	27.9±0.4^{b}
	**LBs**	56.4±2.5^{a}	34.1±0.3^{b}	103.3±2.8^{a}	—	44.3±0.5^{b}	7.1±0.6^{c}
Noon	**Sym**	—	52.4±1.1^{a}	64.2±6.7^{b}	6.5±0.0^{b}	117.1±12.5^{a}	76.2±0.7^{a}
	F	900	4492	114	45199	263	10780
	p	***	***	***	***	***	***
	**Host**	27.1±4.1^{b}	10.1±0.8^{c}	27.0±2.7^{c}	12.6±0.2^{a}	19.1±0.4^{c}	30.5±0.2^{b}
	**LBs**	83.5±2.6^{a}	28.4±1.1^{b}	120.2±1.6^{a}	—	68.7±4.6^{b}	13.5±1.1^{c}
Sunset	**Sym**	—	55.9±1.9^{a}	76.6±1.3^{b}	7.3±0.4^{b}	106.9±11.1^{a}	90.0±3.3^{a}
	F	1414	1753	3361	3819	241	1201
	p	***	***	***	***	***	***
	**Host**	23.2±3.7^{b}	10.0±0.7^{c}	20.2±2.2^{b}	18.7±1.2^{a}	29.7±1.0^{b}	40.6±1.9^{b}
	**LBs**	61.5±3.4^{a}	30.0±1.8^{b}	51.6±0.4^{a}	—	51.0±3.1^{a}	8.2±1.3^{c}
Midnight	**Sym**	—	78.7±6.6^{a}	28.7±6.2^{b}	6.8±0.1^{b}	52.7±1.7^{a}	71.0±0.1^{a}
	F	688	478	110	1039	224	1650
	p	***	***	***	***	***	***
	**Host**	*	0.09	**	*	*	*
Significant level across time *(p)*	**LBs**	**	0.05	**	—	*	*
	**Sym**	—	*	*	*	*	*

Table S3. Concentrations of fatty acids (FAs) within the **total lipids** pool among coral host coral gastrodermal cells (Host), lipid bodies (LBs), and *in hospite Symbiodinium* (Sym) across the diel cycle. Data were analyzed with ANOVA followed by Duncan's multiple-range procedures to determine the effect of cellular compartment for each of 17 FAs (*$p<0.05$, ** $p<0.005$, *** $p<0.001$), and Kruskal-Wallis tests were used to determine the effect of sampling time. Letters adjacent to values (concentration±SD) represent statistically significant differences across sampling times and compartments within a lipid species, as determined by Mann-Whitney post-hoc *U* tests ($p<0.05$). "—" = not detected. SFAs = saturated fatty acids. PUFAs = polyunsaturated fatty acids.

	Concentration (ng/μg protein)																				Significant level across time *(p)*		
Time\	Sunrise					Noon					Sunset					Midnight							
Fatty acids	**Host**	**LBs**	**Sym**	F	*p*	**Host**	**LBs**	**Sym**	F	*p*	**Host**	**LBs**	**Sym**	F	*p*	**Host**	**LBs**	**Sym**	F	*p*	**Host**	**LBs**	**Sym**
14:0	4.2±0.8[a]	2.1±0.5[b]	4.5±0.5[a]	13	**	4.5±1.1[b]	2.5±0.3[b]	7.5±0.8[a]	29	***	1.8±0.9[c]	4.4±0.7[b]	11.2±1.3[a]	66	***	3.6±1.0[ab]	2.9±0.4[b]	4.8±0.1[a]	6	*	0.07	*	*
16:0	31.1±2.9[a]	17.5±1.7[b]	26.8±0.5[a]	39	***	31.5±3.1	35.6±1.6	34.5±0.4	3	0.11	19.9±1.4[c]	55.5±5.1[a]	47.3±0.1[b]	114	***	28.2±2.8[b]	42.2±2.1[a]	23.1±0.4[b]	71	***	*	*	*
18:0	29.6±2.3[a]	13.5±0.6[b]	27.8±0.8[a]	111	***	28.7±2.0	26.1±2.7	30.5±0.4	4	0.08	18.8±0.6[b]	33.1±2.7[a]	35.8±0.9[a]	91	***	24.0±1.1[b]	29.1±3.1[a]	28.0±0.3[ab]	6	*	*	*	*
20:0	1.9±0.6[a]	0.3±0.0[b]	—	23	***	1.8±0.5[a]	0.7±0.1[b]	—	27	***	1.6±0.4[a]	1.0±0.2[b]	—	35	***	1.8±0.4[a]	0.9±0.1[b]	—	46	***	0.95	*	—
22:0	1.8±0.7[a]	—	1.8±0.4[a]	19	***	1.9±0.7[a]	—	2.5±0.5[a]	20	***	2.6±0.4[b]	—	4.5±0.9[a]	48	***	2.0±0.7[a]	—	2.1±0.4[a]	20	***	0.50	—	0.06
16:1 n-7	—	1.5±0.3[a]	2.3±0.6[a]	29	***	—	1.9±0.4[a]	1.5±0.3[a]	39	***	—	2.4±0.5[a]	2.7±0.3[a]	63	***	—	0.6±0.2[b]	1.9±0.4[a]	41	***	—	*	0.09
20:2 n-9	0.4±0.0[b]	—	0.6±0.1[a]	201	***	0.5±0.0[a]	—	0.5±0.1[a]	154	***	0.6±0.0[b]	—	1.3±0.1[a]	639	***	0.4±0.0[b]	—	0.8±0.1[a]	79	***	*	—	*
22:1 n-9	0.5±0.0[b]	2.6±1.0[a]	0.3±0.1[b]	16	***	0.4±0.1[b]	3.5±1.0[a]	0.4±0.1[b]	28	***	0.3±0.1[b]	4.6±0.9[a]	1.1±0.0[b]	63	***	0.8±0.1[b]	3.8±1.0[a]	0.2±0.1[b]	34	***	*	0.15	*
18:1 n-9	1.7±0.1[b]	14.6±3.3[a]	1.6±0.2[b]	46	***	1.7±0.1[b]	12.5±1.7[a]	3.2±0.1[b]	104	***	1.5±0.2[c]	11.5±1.2[a]	4.3±0.1[b]	158	***	1.2±0.1[b]	4.9±1.3[a]	2.0±0.1[b]	20	***	*	*	*
18:2 n-6	7.0±1.4[a]	1.1±0.2[b]	3.0±0.5[b]	37	***	5.7±0.9[a]	2.7±0.7[b]	1.8±0.4[b]	23	***	6.2±0.6[a]	2.9±0.3[c]	4.2±0.3[b]	43	***	4.1±1.0[a]	1.9±0.1[b]	2.1±0.4[b]	12	**	0.06	*	*
18:3 n-6	1.5±0.2[b]	1.5±0.3[b]	20.6±1.2[a]	702	***	1.8±0.1[b]	3.4±0.9[b]	14.6±1.5[a]	139	***	1.9±0.2[c]	5.3±1.5[b]	30.2±0.7[a]	761	***	1.8±0.2[b]	4.7±0.1[b]	18.8±2.1[a]	173	***	0.11	*	*
20:3 n-6	1.5±0.3[a]	1.9±0.5[a]	—	27	***	1.9±0.0[a]	2.8±0.8[a]	—	6	*	1.4±0.2[b]	4.7±1.0[a]	—	53	***	1.4±0.1[b]	3.5±0.7[a]	—	50	***	0.09	0.09	—
20:4 n-6	13.5±3.1[a]	5.5±1.0[b]	—	39	***	14.5±2.4[a]	8.1±1.7[b]	—	56	***	12.5±1.9[a]	7.1±2.2[b]	—	41	***	10.9±2.1[a]	6.2±0.9[b]	—	49	***	0.27	0.32	—
22:4 n-6	4.0±0.9[a]	1.0±0.2[b]	—	44	***	4.4±0.7[a]	1.5±0.3[b]	—	73	***	3.9±0.6[a]	1.4±0.4[b]	—	65	***	3.2±0.6[a]	1.1±0.2[b]	—	55	***	0.25	0.29	—
18:4 n-3	0.3±0.1[b]	0.2±0.0[b]	3.6±0.1[a]	1360	***	0.3±0.1[b]	0.3±0.1[b]	3.2±0.1[a]	2829	***	0.3±0.0[b]	0.3±0.1[b]	5.4±0.5[a]	318	***	0.2±0.0[b]	0.2±0.0[b]	3.5±0.6[a]	100	***	0.29	0.34	*
20:5 n-3	1.4±0.2[b]	0.6±0.1[c]	12.2±0.5[a]	1340	***	1.2±0.1[b]	1.6±0.3[b]	11.6±0.2[a]	2195	***	1.3±0.1[b]	2.0±0.5[b]	17.4±1.6[a]	276	***	1.4±0.2[b]	1.5±0.0[b]	11.7±1.9[a]	87	***	0.55	*	0.06
22:6 n-3	5.2±0.9[b]	6.8±0.9[b]	12.7±0.9[a]	54	***	6.5±1.0[b]	7.0±1.4[b]	11.0±0.1[a]	17	***	5.6±1.1[c]	11.7±2.1[b]	15.5±1.9[a]	25	***	4.2±1.1[c]	6.9±0.7[b]	10.5±1.0[a]	33	***	0.16	0.10	*
SFAs	68.7±6.0[a]	33.3±2.7[b]	61.0±2.0[a]	66	***	68.3±5.6[ab]	64.9±1.3[b]	74.9±0.3[a]	7	*	44.8±1.6[c]	94.0±2.2[b]	98.7±0.5[a]	1024	***	59.5±4.1[b]	75.0±1.3[a]	57.9±0.7[b]	43	***	*	***	***
PUFAs	37.0±5.2[b]	24.3±4.0[b]	57.0±1.9[a]	15	**	38.9±4.1	36.8±7.0	47.7±1.8	2	0.19	35.5±3.8[c]	50.8±7.2[b]	82.2±4.8[a]	60	***	29.4±3.2[b]	34.2±1.9[b]	51.4±6.1[a]	23	***	0.10	**	***
n-7	—	1.5±0.3[a]	2.3±0.6[a]	29	***	—	1.9±0.4[a]	1.5±0.3[a]	39	***	—	2.4±0.5[a]	2.7±0.3[a]	63	***	—	0.6±0.1[b]	1.9±0.4[a]	41	***	—	**	**
n-9	2.6±0.1[b]	4.2±1.2[a]	2.5±0.0[b]	36	***	2.6±0.2[b]	7.3±0.9[a]	4.1±0.2[b]	87	***	2.4±0.2[c]	13.0±0.2[a]	6.7±0.0[b]	510	***	2.3±0.0[b]	7.7±1.8[a]	3.0±0.2[b]	27	***	0.08	***	***
n-6	27.6±4.5[a]	11.0±1.7[b]	23.6±1.7[a]	26	***	28.3±3.0[a]	18.7±4.4[b]	16.4±1.8[b]	11	**	25.9±2.7[ab]	21.4±5.1[b]	34.4±1.0[a]	11	**	21.3±2.2	17.4±0.6	21.0±2.4	4	0.08	0.11	*	***
n-3	6.8±0.9[b]	7.6±1.1[b]	28.6±0.3[a]	624	***	8.1±1.0[b]	8.9±1.8[b]	25.7±0.2[a]	212	***	7.1±1.1[b]	14.0±2.6[b]	38.3±3.9[a]	103	***	5.8±1.0[b]	8.6±0.6[b]	25.6±3.5[a]	78	***	0.12	*	**

Table S4. Concentrations of **TAGs** amongst host coral gastrodermal cells (Host), lipid bodies (LBs), and *in hospite Symbiodinium* (Sym) across the diel cycle. Data were analyzed with ANOVA followed by Duncan's multiple-range procedures to determine the effect of cellular compartment for each of 17 fatty acids (*$p<0.05$, **$p<0.005$, ***$p<0.001$), and Kruskal-Wallis tests were used to determine the effect of sampling time. Letters adjacent to values (concentration±SD) represent statistically significant differences across sampling times and compartment within a lipid species, as determined by Mann-Whitney post-hoc U tests ($p<0.05$). "—" = not detected. SFAs = saturated fatty acids. PUFAs = polyunsaturated fatty acids.

	Concentration (ng/µg protein)																				Significant level across time *(p)*		
Time\	Sunrise					Noon					Sunset					Midnight							
Fatty acids	**Host**	**LBs**	**Sym**	F	*p*	**Host**	**LBs**	**Sym**	F	*p*	**Host**	**LBs**	**Sym**	F	*p*	**Host**	**LBs**	**Sym**	F	*p*	**Host**	**LBs**	**Sym**
14:0	0.6±0.0^{b}	0.7±0.1^{b}	3.6±0.9^{a}	29	***	0.6±0.1^{b}	0.8±0.1^{b}	1.7±0.3^{a}	30	***	0.5±0.0^{b}	1.2±0.2^{b}	2.4±0.5^{a}	29	***	0.3±0.0^{c}	1.0±0.2^{a}	0.6±0.1^{b}	30	***	*	*	*
16:0	12.0±0.3^{a}	10.5±0.4^{a}	10.5±1.0^{a}	5	*	8.9±0.8^{b}	27.7±3.1^{a}	7.0±0.7^{b}	111	***	8.0±0.4^{b}	22.9±3.7^{a}	10.7±0.3^{b}	41	***	4.7±0.9^{b}	20.7±3.1^{a}	3.6±0.3^{b}	78	***	*	*	*
18:0	10.4±1.0^{ab}	8.7±0.8^{b}	13.1±1.7^{a}	9	**	6.2±0.3^{b}	18.8±2.1^{a}	5.9±0.9^{b}	92	***	6.7±0.3^{c}	14.7±1.2^{a}	11.5±0.3^{b}	86	***	3.3±0.3^{b}	12.5±1.3^{a}	3.0±0.5^{b}	128	***	*	*	*
20:0	0.6±0.1^{a}	0.3±0.0^{b}	—	93	***	0.2±0.1^{b}	0.7±0.0^{a}	—	204	***	0.2±0.1^{a}	0.2±0.1^{a}	—	18	***	0.1±0.0^{b}	0.5±0.1^{a}	—	76	***	*	*	—
22:0	0.6±0.1	—	—	101	***	0.4±0.1	—	—	62	***	0.3±0.1	—	—	23	***	0.2±0.0	—	—	55	***	*	—	—
16:1 n-7	—	0.1±0.0^{b}	0.1±0.0^{a}	22	***	—	0.4±0.0^{a}	0.3±0.1^{a}	28	***	—	0.3±0.1^{b}	0.6±0.1^{a}	45	***	—	0.2±0.0^{a}	0.1±0.0^{b}	51	***	—	*	*
20:2 n-9	—	—	—	—	—	—	—	—	—	—	—	—	—	—	—	—	—	—	—	—	—	—	—
22:1 n-9	0.4±0.1^{b}	1.5±0.2^{a}	0.6±0.1^{b}	48	***	1.3±0.4^{a}	1.4±0.3^{a}	0.5±0.2^{b}	9	*	0.9±0.2^{b}	1.6±0.2^{a}	1.2±0.3^{ab}	6	*	0.3±0.1^{b}	1.6±0.3^{a}	0.2±0.1^{b}	63	***	*	0.64	*
18:1 n-9	1.7±0.2	1.7±0.2	1.5±0.2	1	0.40	4.3±1.0	3.5±0.9	2.8±0.1	3	0.12	1.4±0.2^{c}	6.1±0.8^{a}	4.4±0.7^{b}	40	***	0.7±0.2^{b}	2.5±0.4^{a}	0.9±0.1^{b}	32	***	*	*	*
18:2 n-6	0.2±0.0^{b}	0.3±0.0^{a}	0.1±0.0^{c}	42	***	0.1±0.0^{b}	0.3±0.0^{a}	0.2±0.0^{b}	32	***	0.3±0.0^{b}	0.4±0.1^{ab}	0.6±0.1^{a}	12	**	0.1±0.0^{c}	0.5±0.1^{a}	0.2±0.0^{b}	58	***	*	*	*
18:3 n-6	0.1±0.0^{b}	0.3±0.0^{a}	0.3±0.0^{a}	32	***	0.5±0.1^{b}	0.5±0.1^{b}	0.7±0.1^{a}	7	*	0.9±0.1^{b}	0.4±0.1^{b}	3.2±0.4^{a}	133	***	0.5±0.1	0.5±0.1	0.6±0.1	1	0.44	*	*	*
20:3 n-6	0.9±0.1^{a}	0.8±0.2^{a}	—	82	***	3.5±0.9^{a}	1.7±0.3^{b}	—	30	***	1.7±0.5^{b}	6.6±0.9^{a}	—	100	***	1.3±0.3^{a}	1.2±0.0^{a}	—	54	***	*	*	—
20:4 n-6	0.9±0.0^{b}	1.7±0.1^{a}	—	979	***	4.3±0.1^{a}	3.3±0.2^{b}	—	1413	***	3.0±0.8^{b}	7.9±0.4^{a}	—	184	***	1.6±0.0^{b}	2.4±0.1^{a}	—	1012	***	*	*	—
22:4 n-6	0.3±0.0^{b}	0.5±0.0^{a}	—	979	***	1.3±0.0^{a}	1.0±0.0^{b}	—	1413	***	0.9±0.2^{b}	2.4±0.1^{a}	—	184	***	0.5±0.0^{b}	0.7±0.0^{a}	—	1012	***	*	*	—
18:4 n-3	0.7±0.0^{b}	1.2±0.1^{a}	0.1±0.0^{c}	781	***	1.0±0.0^{a}	0.7±0.0^{b}	0.2±0.0^{c}	771	***	0.7±0.2^{b}	1.7±0.1^{a}	0.5±0.1^{b}	98	***	0.3±0.0^{b}	0.5±0.0^{a}	0.1±0.0^{c}	490	***	*	*	*
20:5 n-3	—	0.1±0.1^{b}	0.3±0.1^{a}	20	***	—	0.9±0.0^{a}	0.8±0.0^{a}	936	***	—	0.9±0.0^{b}	1.6±0.2^{a}	118	***	—	0.7±0.0^{a}	0.4±0.0^{b}	331	***	—	*	*
22:6 n-3	0.5±0.1^{b}	0.8±0.1^{a}	0.3±0.1^{b}	25	***	3.4±1.0^{b}	1.8±0.3^{b}	0.7±0.1^{a}	16	***	1.9±0.5^{b}	5.1±1.2^{a}	1.4±0.2^{b}	21	***	1.3±0.2^{a}	1.6±0.2^{a}	0.2±0.0^{b}	47	***	*	*	*
SFAs	24.2±1.3^{a}	20.3±1.0^{b}	27.1±1.9^{a}	17	***	16.2±0.5^{b}	48.0±1.0^{a}	14.6±1.8^{b}	697	***	15.7±0.3^{c}	39.0±2.2^{a}	24.7±0.9^{b}	223	***	8.6±0.6^{b}	34.7±1.8^{a}	7.2±0.8^{b}	486	***	*	*	*
PUFAs	5.6±0.5^{b}	9.1±0.4^{a}	3.2±0.1^{c}	179	***	19.8±3.0^{a}	15.4±1.0^{a}	6.2±0.2^{b}	43	***	11.8±1.4^{b}	33.5±1.1^{a}	13.5±1.3^{b}	267	***	6.5±0.6^{b}	12.4±0.3^{a}	2.8±0.1^{c}	500	***	*	*	*
n-7	—	0.1±0.0^{a}	0.1±0.0^{a}	22	***	—	0.4±0.0^{a}	0.3±0.1^{a}	28	***	—	0.3±0.1^{b}	0.6±0.1^{a}	45	***	—	0.2±0.0^{b}	0.1±0.0^{a}	51	***	—	*	*
n-9	2.1±0.4^{b}	3.2±0.2^{a}	2.0±0.2^{b}	22	***	5.7±1.1^{a}	4.9±1.0^{ab}	3.3±0.2^{b}	6	*	2.4±0.2^{c}	7.8±0.6^{a}	5.6±1.1^{b}	44	***	0.9±0.2^{b}	4.0±0.4^{a}	1.2±0.2^{b}	118	***	*	*	*
n-6	2.4±0.1^{b}	3.7±0.2^{a}	0.4±0.0^{c}	739	***	9.8±1.0^{a}	6.8±0.3^{b}	0.9±0.1^{c}	150	***	6.9±1.0^{b}	17.7±0.9^{a}	3.8±0.4^{c}	239	***	3.9±0.3^{b}	5.4±0.2^{a}	0.8±0.1^{c}	323	***	*	*	*
n-3	1.1±0.1^{b}	2.1±0.1^{a}	0.6±0.1^{c}	114	***	4.4±1.0^{a}	3.4±0.2^{a}	1.7±0.1^{b}	15	***	2.6±0.6^{b}	7.7±1.1^{a}	3.5±0.5^{b}	40	***	1.6±0.2^{b}	2.8±0.2^{a}	0.7±0.1^{c}	107	***	*	*	*

Table S5. Concentrations of **SEs** amongst host coral gastrodermal cells (Host), lipid bodies (LBs), and *in hospite Symbiodinium* (Sym) across the diel cycle. Data were analyzed with ANOVA followed by Duncan's multiple-range procedures to determine the effect of cellular compartment for each of 17 fatty acids (*p<0.05, ** p<0.005, *** p<0.001), and Kruskal-Wallis tests were used to determine the effect of sampling time. Letters adjacent to values (concentration±SD) represent statistically significant differences across sampling times and compartment within a lipid species, as determined by Mann-Whitney post-hoc U tests (p<0.05). "—" = not detected. SFAs = saturated fatty acids. PUFAs = polyunsaturated fatty acids.

Time\	Concentration (ng/µg protein)																				Significant level across time *(p)*		
	Sunrise					Noon					Sunset					Midnight							
Fatty acids	**Host**	**LBs**	**Sym**	F	*p*	**Host**	**LBs**	**Sym**	F	*p*	**Host**	**LBs**	**Sym**	F	*p*	**Host**	**LBs**	**Sym**	F	*p*	**Host**	**LBs**	**Sym**
14:0	0.6±0.0^{a}	0.4±0.1^{c}	0.7±0.0^{a}	60	***	0.6±0.1^{a}	0.4±0.0^{b}	0.1±0.0^{c}	145	***	0.3±0.1^{b}	0.3±0.0^{b}	1.5±0.3^{a}	64	***	0.2±0.0^{b}	0.5±0.0^{b}	2.6±0.4^{a}	81	***	*	0.08	*
16:0	4.1±0.0^{b}	4.2±0.4^{b}	11.8±2.0^{a}	44	***	7.4±0.1^{a}	9.3±1.4^{a}	4.1±0.7^{b}	26	***	3.0±0.3^{c}	4.8±0.1^{b}	8.0±0.8^{a}	77	***	2.4±0.4^{c}	6.5±0.3^{b}	18.9±2.1^{a}	140	***	*	*	*
18:0	3.0±0.9^{b}	3.2±0.5^{b}	10.2±1.7^{a}	40	***	7.5±0.7^{a}	7.7±0.7^{a}	3.6±0.6^{b}	34	***	2.5±0.4^{b}	4.0±0.6^{b}	12.2±1.5^{a}	91	***	2.7±0.8^{c}	8.2±0.6^{b}	18.6±2.1^{a}	111	***	0.09	*	*
20:0	—	—	—	—	—	—	—	—	—	—	—	—	—	—	—	—	—	—	—	—	—	—	—
22:0	—	—	—	—	—	—	—	—	—	—	—	—	—	—	—	—	—	—	—	—	—	—	—
16:1 n-7	—	—	0.4±0.1	108	***	—	—	0.2±0.0	108	***	—	—	0.4±0.1	108	***	—	—	—	—	—	—	—	*
20:2 n-9	—	—	—	—	—	—	—	—	—	—	—	—	—	—	—	—	—	—	—	—	—	—	—
22:1 n-9	1.0±0.2	—	—	77	***	0.3±0.1	—	—	27	***	0.1±0.0	—	—	26	***	0.4±0.1	—	—	59	***	*	—	—
18:1 n-9	2.5±0.3	1.8±0.9	2.0±0.2	1	0.44	2.0±0.3^{a}	1.4±0.4^{a}	0.6±0.1^{b}	17	***	1.1±0.6^{b}	1.0±0.2^{b}	3.0±0.5^{a}	18	***	0.5±0.0^{b}	1.0±0.1^{b}	8.4±1.4^{a}	90	***	*	0.46	*
18:2 n-6	1.2±0.2^{ab}	1.1±0.3^{b}	1.7±0.1^{a}	8	*	0.5±0.1^{b}	1.3±0.3^{a}	0.5±0.1^{b}	21	***	0.8±0.1^{a}	0.2±0.0^{c}	0.5±0.1^{b}	32	***	0.4±0.1^{a}	0.2±0.0^{b}	—	23	***	*	*	*
18:3 n-6	—	0.8±0.2^{b}	6.6±1.1^{a}	95	***	—	0.2±0.0^{b}	4.1±0.7^{a}	104	***	—	—	3.7±0.6	108	***	—	—	2.5±0.4	108	***	—	*	*
20:3 n-6	—	0.5±0.1	—	73	***	—	0.4±0.1	—	64	***	—	0.2±0.0	—	109	***	—	0.2±0.0	—	130	***	—	*	—
20:4 n-6	0.9±0.1^{a}	0.6±0.2^{b}	—	55	***	0.7±0.2^{a}	0.3±0.1^{b}	—	27	***	0.4±0.1^{b}	0.2±0.1^{a}	—	21	***	—	0.2±0.0	—	83	***	*	*	—
22:4 n-6	—	—	—	—	—	—	—	—	—	—	—	—	—	—	—	—	—	—	—	—	—	—	—
18:4 n-3	—	—	—	—	—	—	—	—	—	—	—	—	—	—	—	—	—	—	—	—	—	—	—
20:5 n-3	0.3±0.0^{b}	—	1.0±0.2^{a}	86	***	0.3±0.1^{a}	—	0.3±0.0^{a}	35	***	0.3±0.1^{b}	—	0.9±0.2^{a}	72	***	0.3±0.1^{b}	—	0.9±0.1^{a}	57	***	0.96	—	0.08
22:6 n-3	1.1±0.2^{a}	1.2±0.4^{a}	0.6±0.1^{a}	4	0.07	0.9±0.1^{a}	0.5±0.2^{b}	0.5±0.1^{b}	11	*	0.6±0.2^{ab}	0.4±0.1^{b}	0.8±0.1^{a}	5	0.05	0.2±0.1^{b}	0.3±0.0^{b}	2.1±0.4^{a}	80	***	*	*	*
SFAs	7.7±0.9^{b}	7.7±0.1^{b}	22.7±3.6^{a}	48	***	15.5±0.8^{a}	17.4±2.1^{a}	7.8±1.3^{b}	33	***	5.7±0.5^{b}	9.1±0.5^{b}	21.7±2.6^{a}	89	***	5.3±1.2^{c}	15.2±1.0^{b}	30.1±4.6^{a}	122	***	*	*	*
PUFAs	6.9±0.7^{b}	6.0±1.1^{b}	13.5±1.7^{a}	23	***	4.7±0.5^{ab}	4.0±0.2^{b}	6.6±1.1^{a}	7	*	3.4±0.5^{b}	2.1±0.1^{b}	10.8±1.5^{a}	50	***	1.8±0.3^{b}	1.8±0.1^{b}	18.2±1.0^{a}	81	***	*	*	*
n-7	—	—	0.4±0.1	108	***	—	—	0.2±0.0	108	***	—	—	0.4±0.1	108	***	—	—	—	—	—	—	—	*
n-9	3.4±0.4^{a}	1.8±0.9^{b}	1.4±0.1^{ab}	7	*	2.3±0.2^{a}	1.4±0.4^{b}	0.5±0.1^{c}	30	***	1.2±0.6^{b}	1.0±0.2^{b}	2.2±0.4^{a}	18	***	0.9±0.1^{b}	1.0±0.1^{b}	2.9±0.3^{a}	85	***	*	0.41	*
n-6	2.2±0.2^{b}	3.0±0.7^{b}	9.8±1.2^{a}	53	***	1.2±0.2^{b}	2.2±0.2^{b}	5.0±0.8^{a}	40	***	1.2±0.2^{b}	0.7±0.1^{b}	5.9±0.8^{a}	59	***	0.4±0.1^{b}	0.5±0.1^{b}	12.1±0.7^{a}	67	***	*	*	*
n-3	1.4±0.2	1.2±0.4	2.0±0.3	2	0.24	1.2±0.1^{a}	0.5±0.2^{b}	1.0±0.2^{b}	22	***	0.9±0.2^{b}	0.4±0.1^{b}	2.4±0.3^{a}	31	***	0.5±0.2^{b}	0.3±0.0^{b}	3.2±0.5^{a}	74	***	*	*	*

Table S6. Concentrations of **FFAs** amongst host coral gastrodermal cells (Host), lipid bodies (LBs), and *in hospite Symbiodinium* (Sym) across the diel cycle. Data were analyzed with ANOVA followed by Duncan's multiple-range procedures to determine the effect of cellular compartment for each of 17 fatty acids (*p<0.05, **p<0.005, ***p<0.001), and Kruskal-Wallis tests were used to determine the effect of sampling time. Letters adjacent to values (concentration±SD) represent statistically significant differences across sampling times and compartment within a lipid species, as determined by Mann-Whitney post-hoc U tests (p<0.05). "—" = not detected. SFAs = saturated fatty acids. PUFAs = polyunsaturated fatty acids.

Time\	Concentration (ng/µg protein)																				Significant level		
	Sunrise					Noon					Sunset					Midnight					across time *(p)*		
Fatty acids	**Host**	**LBs**	**Sym**	F	*p*	**Host**	**LBs**	**Sym**	F	*p*	**Host**	**LBs**	**Sym**	F	*p*	**Host**	**LBs**	**Sym**	F	*p*	**Host**	**LBs**	**Sym**
14:0	1.2±0.3^{a}	0.5±0.1^{b}	0.9±0.0^{a}	16	***	0.6±0.1^{b}	0.4±0.1^{b}	6.0±0.1^{a}	2290	***	0.4±0.0^{b}	0.6±0.0^{b}	4.4±1.3^{a}	25	***	0.8±0.1^{b}	0.7±0.1^{b}	1.5±0.1^{a}	55	***	*	*	*
16:0	9.0±0.8^{a}	5.2±0.6^{b}	7.8±0.7^{a}	22	***	6.9±0.4^{c}	8.1±1.1^{b}	27.0±0.8^{a}	863	***	4.9±0.5^{b}	15.3±1.7^{a}	18.3±1.1^{a}	188	***	7.6±0.6^{b}	11.2±1.3^{a}	7.9±0.5^{b}	54	***	*	*	*
18:0	9.2±0.7^{a}	5.5±0.6^{b}	4.4±0.1^{c}	72	***	7.9±0.7^{b}	5.9±0.6^{b}	21.9±3.0^{a}	63	***	5.3±0.7^{c}	11.9±0.9^{a}	9.1±0.1^{b}	47	***	12.0±1.3^{a}	9.6±1.0^{b}	5.4±0.7^{c}	40	***	*	*	*
20:0	0.2±0.0	—	—	1197	***	0.1±0.0	—	—	26	***	0.2±0.0	—	—	1201	***	0.1±0.0	—	—	28	***	*	—	—
22:0	0.5±0.1^{a}	—	0.3±0.1^{b}	49	***	0.4±0.1^{b}	—	0.6±0.1^{a}	52	***	0.4±0.1^{a}	—	0.5±0.1^{a}	67	***	0.5±0.0^{a}	—	0.3±0.1^{b}	164	***	0.35	—	*
16:1 n-7	—	0.4±0.1^{a}	0.6±0.0^{b}	459	***	—	1.0±0.2^{a}	0.2±0.0^{b}	46	***	—	1.8±0.1^{b}	1.0±0.1^{a}	71	***	—	0.2±0.0^{b}	0.4±0.1^{a}	53	***	—	*	*
20:2 n-9	—	—	—	—	—	—	—	—	—	—	—	—	—	—	—	—	—	—	—	—	—	—	—
22:1 n-9	1.6±0.3^{b}	2.6±0.6^{a}	—	31	***	1.0±0.1^{a}	2.0±0.8^{a}	—	14	**	0.3±0.0^{b}	1.2±0.2^{a}	—	91	***	0.8±0.0^{b}	4.5±0.1^{a}	—	135	***	*	*	—
18:1 n-9	2.7±0.6^{a}	3.1±1.1^{a}	1.4±0.2^{a}	5	0.05	1.4±0.1^{b}	3.0±1.6^{b}	5.5±0.3^{a}	15	**	0.9±0.2^{b}	2.4±0.5^{b}	7.6±1.1^{a}	77	***	1.0±0.1^{b}	4.8±0.9^{a}	1.1±0.2^{b}	56	***	*	0.24	*
18:2 n-6	0.4±0.1^{b}	1.7±0.4^{a}	1.3±0.2^{a}	17	***	0.2±0.1^{b}	0.4±0.1^{b}	1.5±0.1^{a}	97	***	0.1±0.0^{b}	0.4±0.1^{b}	2.5±0.2^{a}	264	***	0.1±0.0^{c}	1.8±0.1^{a}	0.9±0.0^{b}	919	***	*	*	*
18:3 n-6	—	—	10.4±0.7	587	***	—	—	5.2±0.5	356	***	—	—	11.4±1.0	381	***	—	—	6.5±0.4	797	***	—	—	*
20:3 n-6	—	—	—	—	—	—	—	—	—	—	—	—	—	—	—	—	—	—	—	—	—	—	—
20:4 n-6	0.7±0.1^{b}	3.4±1.0^{a}	—	30	***	0.5±0.1^{b}	2.8±0.9^{a}	—	24	***	0.7±0.1^{b}	1.6±0.2^{a}	—	119	***	0.4±0.1^{b}	5.0±0.5^{a}	—	118	***	*	*	—
22:4 n-6	0.5±0.1^{a}	0.8±0.2^{a}	—	25	***	0.4±0.1^{a}	0.6±0.2^{a}	—	20	***	0.5±0.0^{a}	0.4±0.0^{b}	—	155	***	0.3±0.1^{b}	0.9±0.1^{a}	—	92	***	*	*	—
18:4 n-3	—	—	2.3±0.3	237	***	—	—	1.0±0.1	643	***	—	—	2.4±0.3	173	***	—	—	1.1±0.1	257	***	—	—	*
20:5 n-3	—	0.5±0.1^{b}	5.5±0.6^{a}	211	***	—	0.5±0.1^{b}	2.7±0.2^{a}	368	***	—	0.5±0.1^{b}	5.3±0.7^{a}	154	***	—	0.3±0.0^{b}	2.6±0.3^{a}	233	***	—	0.08	*
22:6 n-3	0.7±0.2^{b}	1.7±0.6^{b}	5.3±0.7^{a}	61	***	0.4±0.1^{c}	1.4±0.4^{b}	2.4±0.3^{a}	32	***	0.4±0.1^{b}	1.5±0.3^{b}	3.8±0.7^{a}	44	***	0.2±0.0^{b}	2.9±0.5^{a}	2.4±0.3^{a}	147	***	*	0.08	*
SFAs	20.1±1.8^{a}	11.2±1.3^{b}	13.5±0.7^{b}	35	***	15.9±1.0^{b}	16.0±1.0^{b}	55.5±2.7^{a}	507	***	11.2±1.3^{b}	30.2±1.0^{a}	32.3±2.4^{a}	143	***	21.0±1.6^{a}	20.6±0.9^{a}	15.0±0.9^{b}	25	***	*	*	*
PUFAs	6.6±1.1^{c}	15.0±2.4^{b}	26.7±2.3^{a}	76	***	3.9±0.5^{c}	11.9±1.7^{b}	18.5±1.0^{a}	117	***	2.9±0.2^{c}	8.8±0.8^{b}	34.0±3.6^{a}	182	***	2.7±0.1^{c}	17.8±1.5^{a}	15.0±0.3^{b}	232	***	*	*	*
n-7	—	1.3±0.1^{a}	0.6±0.0^{b}	459	***	—	1.0±0.2^{a}	0.2±0.0^{b}	46	***	—	0.8±0.1^{b}	1.0±0.1^{a}	71	***	—	0.2±0.0^{b}	0.4±0.1^{a}	53	***	—	*	*
n-9	4.3±0.6^{a}	5.7±1.0^{a}	1.4±0.2^{b}	32	***	2.4±0.2^{b}	5.1±0.9^{a}	5.5±0.3^{a}	30	***	1.2±0.2^{c}	3.6±0.5^{b}	7.6±1.1^{a}	65	***	1.7±0.1^{b}	7.9±0.9^{a}	1.1±0.2^{b}	157	***	*	*	*
n-6	1.6±0.3^{c}	5.9±1.4^{b}	11.6±1.0^{a}	80	***	1.1±0.2^{c}	3.8±1.2^{b}	6.7±0.6^{a}	37	***	1.3±0.1^{b}	2.4±0.3^{b}	13.9±1.2^{a}	272	***	0.7±0.1^{b}	6.8±0.8^{a}	7.4±0.4^{a}	152	***	*	*	*
n-3	0.7±0.2^{b}	2.1±0.7^{b}	13.1±1.6^{a}	139	***	0.4±0.1^{c}	2.0±0.5^{b}	6.1±0.4^{a}	174	***	0.4±0.1^{b}	2.0±0.3^{b}	11.5±1.6^{a}	128	***	0.2±0.0^{c}	2.9±0.1^{b}	6.1±0.7^{a}	150	***	*	0.14	*

Table S7. Concentrations of **PLs** amongst host coral gastrodermal cells (Host), lipid bodies (LBs), and *in hospite Symbiodinium* (Sym) across the diel cycle. Data were analyzed with ANOVA followed by Duncan's multiple-range procedures to determine the effect of cellular compartment for each of 17 fatty acids (**p*<0.05, ***p*<0.005, ****p*<0.001), and Kruskal-Wallis tests were used to determine the effect of sampling time. Letters adjacent to values (concentration±SD) represent statistically significant differences across sampling times and compartment within a lipid species, as determined by Mann-Whitney post-hoc *U* tests (*p*<0.05). "—" = not detected. SFAs = saturated fatty acids. PUFAs = polyunsaturated fatty acids.

	Concentration (ng/μg protein)																				Significant level across time (*p*)		
Time\	Sunrise					Noon					Sunset					Midnight							
Fatty acids	**Host**	**LBs**	**Sym**	F	*p*	**Host**	**LBs**	**Sym**	F	*p*	**Host**	**LBs**	**Sym**	F	*p*	**Host**	**LBs**	**Sym**	F	*p*	**Host**	**LBs**	**Sym**
14:0	0.5±0.1^{b}	0.5±0.0^{b}	0.8±0.0^{a}	15	**	1.8±0.5^{a}	0.4±0.0^{b}	0.8±0.0^{b}	19	***	0.3±0.1^{c}	0.7±0.0^{b}	1.4±0.1^{a}	217	***	1.0±0.2^{b}	0.3±0.0^{b}	3.0±0.7^{a}	38	***	*	*	*
16:0	6.9±0.8^{a}	3.2±0.3^{b}	6.8±0.4^{a}	47	***	7.9±0.3^{a}	4.3±0.5^{b}	10.0±1.4^{a}	31	***	4.3±0.3^{c}	10.7±0.6^{b}	15.8±1.5^{a}	110	***	10.3±0.4^{a}	3.6±0.4^{b}	10.4±0.6^{a}	216	***	*	*	*
18:0	7.8±0.8^{a}	3.8±0.4^{b}	3.1±0.1^{b}	76	***	8.7±0.6^{a}	3.4±0.4^{b}	3.0±0.4^{b}	143	***	3.6±0.1^{b}	5.2±0.3^{b}	10.0±1.1^{a}	81	***	8.9±0.5^{a}	2.8±0.3^{c}	5.6±0.4^{b}	148	***	*	*	*
20:0	0.4±0.1	—	—	43	***	0.4±0.1	—	—	73	***	0.3±0.1	—	—	46	***	0.5±0.1	—	—	59	***	0.25	—	—
22:0	0.5±0.1^{a}	—	0.6±0.1^{a}	53	***	0.5±0.1^{a}	—	0.3±0.0^{b}	64	***	0.5±0.1^{b}	—	0.9±0.1^{a}	83	***	0.6±0.1^{b}	—	1.3±0.2^{a}	82	***	0.53	—	*
16:1 n-7	—	—	0.1±0.0	28	***	—	—	0.2±0.1	27	***	—	—	0.2±0.0	147	***	—	—	0.2±0.1	27	***	—	—	0.11
20:2 n-9	0.2±0.0^{b}	—	0.4±0.1^{a}	56	***	0.4±0.0^{a}	—	0.4±0.0^{a}	112	***	0.5±0.0^{b}	—	0.9±0.1^{a}	444	***	0.2±0.0^{b}	—	0.4±0.1^{a}	38	***	*	—	0.07
22:1 n-9	—	2.0±0.2	—	266	***	—	2.2±0.2	—	245	***	—	2.6±0.1	—	939	***	—	1.8±0.2	—	266	***	—	0.05	—
18:1 n-9	2.9±0.3^{a}	0.1±0.0^{c}	0.8±0.1^{b}	218	***	1.2±0.1^{a}	0.3±0.0^{c}	0.7±0.1^{b}	207	***	0.6±0.1^{b}	0.3±0.0^{b}	2.7±0.6^{a}	35	***	1.3±0.1^{a}	0.2±0.0^{b}	1.6±0.2^{a}	107	***	*	*	*
18:2 n-6	0.6±0.1^{a}	—	0.4±0.1^{b}	46	***	0.4±0.1^{b}	—	0.6±0.0^{a}	109	***	0.2±0.1^{b}	—	0.6±0.1^{a}	74	***	0.4±0.1^{b}	—	0.6±0.1^{a}	61	***	*	—	0.07
18:3 n-6	0.5±0.2^{b}	—	5.9±0.8^{a}	150	***	0.4±0.1^{b}	—	7.6±1.2^{a}	109	***	0.3±0.1^{b}	—	7.5±0.9^{a}	181	***	0.5±0.1^{b}	—	5.5±0.6^{a}	252	***	0.36	—	0.07
20:3 n-6	0.9±0.2	—	—	70	***	1.0±0.2	—	—	77	***	1.1±0.2	—	—	77	***	0.7±0.1	—	—	165	***	0.06	—	—
20:4 n-6	7.6±0.3^{a}	3.2±0.3^{b}	—	629	***	3.5±0.3^{a}	3.2±0.4^{a}	—	158	***	5.4±0.2^{a}	5.3±0.3^{a}	—	660	***	6.3±0.7^{a}	4.3±0.5^{b}	—	126	***	*	*	—
22:4 n-6	3.2±0.1^{a}	0.7±0.1^{b}	—	1112	***	1.5±0.1^{a}	0.7±0.1^{b}	—	218	***	2.2±0.1^{a}	1.2±0.1^{b}	—	977	***	2.7±0.3^{a}	1.0±0.1^{b}	—	154	***	*	*	—
18:4 n-3	—	—	1.5±0.2	294	***	—	—	2.3±0.3	252	***	—	—	2.8±0.5	107	***	—	—	1.6±0.2	225	***	—	—	*
20:5 n-3	2.9±0.8^{b}	—	5.1±0.5^{a}	67	***	2.6±0.6^{b}	—	8.3±0.9^{a}	139	***	2.5±1.0^{b}	0.5±0.0^{b}	8.9±1.5^{a}	56	***	2.4±0.6^{b}	0.1±0.0^{c}	5.4±0.6^{a}	79	***	0.66	*	*
22:6 n-3	3.0±0.0^{b}	0.3±0.0^{c}	3.9±0.5^{a}	152	***	2.5±0.4^{b}	0.4±0.0^{c}	4.6±0.2^{a}	232	***	3.6±0.9^{b}	0.6±0.0^{c}	7.7±1.4^{a}	41	***	3.4±0.9^{a}	0.6±0.1^{b}	4.9±0.7^{a}	33	***	0.19	*	*
SFAs	16.1±1.5^{a}	7.5±0.8^{c}	11.3±0.3^{b}	59	***	19.4±1.4^{a}	8.2±0.9^{c}	14.1±1.9^{b}	44	***	8.9±0.4^{c}	16.6±0.9^{b}	28.2±2.7^{a}	99	***	21.2±0.5^{a}	6.6±0.7^{b}	20.3±0.9^{a}	373	***	*	*	*
PUFAs	21.9±0.7^{a}	6.3±0.7^{c}	18.2±1.1^{b}	264	***	13.6±1.3^{b}	6.8±0.8^{c}	24.6±2.7^{a}	75	***	16.4±1.4^{b}	10.6±0.6^{c}	31.2±3.7^{a}	63	***	17.8±0.9^{a}	8.1±0.9^{b}	20.2±2.4^{a}	52	***	*	*	*
n-7	—	—	0.1±0.0	28	***	—	—	0.2±0.1	27	***	—	—	0.2±0.0	147	***	—	—	0.2±0.1	27	***	—	—	0.11
n-9	3.2±0.2^{a}	2.1±0.2^{b}	1.2±0.1^{c}	77	***	1.6±0.0^{b}	2.5±0.3^{a}	1.0±0.1^{c}	60	***	1.1±0.1^{b}	2.9±0.2^{a}	3.5±0.6^{a}	36	***	1.5±0.1^{b}	2.1±0.2^{a}	2.0±0.2^{ab}	7	*	*	0.05	*
n-6	12.8±0.3^{a}	3.9±0.4^{c}	6.4±0.8^{b}	201	***	6.8±0.5^{a}	3.9±0.4^{b}	8.2±1.3^{a}	20	***	9.2±0.2^{a}	6.5±0.4^{b}	8.1±1.0^{ab}	14	**	10.5±1.0^{a}	5.2±0.6^{b}	6.1±0.7^{b}	40	***	*	*	0.05
n-3	5.9±0.8^{b}	0.3±0.0^{c}	10.5±1.1^{a}	133	***	5.2±0.9^{b}	0.4±0.0^{c}	15.2±1.4^{a}	192	***	6.1±1.2^{b}	1.2±0.1^{b}	19.4±3.3^{a}	65	***	5.8±0.4^{b}	0.8±0.1^{c}	12.0±1.5^{a}	115	***	0.63	*	*

Table S8. The PCA eigenvalue scores of dynamic change of total fatty acid pools.

**Fig. 2A**	**Eigenvalue**	**% variance**	**% Cumulative variance**	**Eigenvectors**	**PC 1**	**PC 2**	**PC 3**
**PC 1**	201.118	48.898	48.898	**14:0**	0.040	-0.059	-0.024
**PC 2**	119.183	28.977	77.875	**16:0**	-0.281	0.016	-0.376
**PC 3**	76.901	18.697	96.572	**18:0**	-0.114	-0.166	-0.031
**PC 4**	7.679	1.867	98.439	**20:0**	-0.046	-0.009	0.048
**PC 5**	2.563	0.623	99.062	**22:0**	0.013	-0.064	0.073
**PC 6**	1.779	0.433	99.495	**16:1 n-7**	0.043	0.036	-0.039
**PC 7**	0.794	0.193	99.688	**20:2 n-9**	0.009	-0.017	0.018
**PC 8**	0.436	0.106	99.794	**22:1 n-9**	-0.024	0.105	-0.069
**PC 9**	0.318	0.077	99.871	**18:1 n-9**	0.018	0.512	-0.104
**PC 10**	0.284	0.069	99.940	**18:2 n-6**	-0.075	-0.054	0.195
**PC 11**	0.113	0.028	99.968	**18:3 n-6**	0.408	-0.243	-0.126
**PC 12**	0.099	0.024	99.992	**20:3 n-6**	-0.059	0.068	-0.020
**PC 13**	0.023	0.006	99.997	**20:4 n-6**	-0.309	0.076	0.409
**PC 14**	0.009	0.002	100.000	**22:4 n-6**	-0.088	-0.004	0.141
				**18:4 n-3**	0.082	-0.052	-0.015
				**20:5 n-3**	0.257	-0.178	-0.064
				**22:6 n-3**	0.127	0.032	-0.018

**Fig. 2B**	**Eigenvalue**	**% variance**	**% Cumulative variance**	**Eigenvectors**	**PC 1**	**PC 2**
**PC 1**	83.979	91.935	91.935	**14:0**	-0.064	-0.225
**PC 2**	4.007	4.387	96.322	**16:0**	-0.316	0.316
**PC 3**	1.547	1.694	98.016	**18:0**	-0.218	-0.414
**PC 4**	0.889	0.973	98.989	**20:0**	0.007	0.108
**PC 5**	0.541	0.592	99.581	**22:0**	0.046	0.194
**PC 6**	0.291	0.318	99.899	**16:1 n-7**	0.000	0.000
**PC 7**	0.057	0.063	99.962	**20:2 n-9**	0.014	0.000
**PC 8**	0.023	0.025	99.987	**22:1 n-9**	-0.014	0.004
**PC 9**	0.010	0.011	99.998	**18:1 n-9**	0.018	-0.028
**PC 10**	0.002	0.002	100.000	**18:2 n-6**	0.118	-0.617
				**18:3 n-6**	0.021	0.067
				**20:3 n-6**	0.005	0.041
				**20:4 n-6**	0.200	0.257
				**22:4 n-6**	0.070	0.075
				**18:4 n-3**	0.005	0.007
				**20:5 n-3**	0.007	-0.070
				**22:6 n-3**	0.101	0.285

**Fig. 2C**	**Eigenvalue**	**% variance**	**% Cumulative variance**	**Eigenvectors**	**PC 1**	**PC 2**
**PC 1**	280.195	92.655	92.655	**14:0**	0.003	-0.069
**PC 2**	13.350	4.415	97.070	**16:0**	-0.344	0.346
**PC 3**	5.499	1.819	98.888	**18:0**	-0.168	-0.531
**PC 4**	1.788	0.591	99.479	**20:0**	-0.009	0.016
**PC 5**	0.575	0.190	99.669	**22:0**	0.000	0.000
**PC 6**	0.490	0.162	99.831	**16:1 n-7**	0.030	-0.053
**PC 7**	0.352	0.116	99.948	**20:2 n-9**	0.000	0.000
**PC 8**	0.093	0.031	99.979	**22:1 n-9**	0.020	0.098
**PC 9**	0.053	0.017	99.996	**18:1 n-9**	0.376	-0.366
**PC 10**	0.011	0.004	100.000	**18:2 n-6**	-0.003	0.044
				**18:3 n-6**	-0.042	0.127
				**20:3 n-6**	-0.007	0.123
				**20:4 n-6**	0.068	0.115
				**22:4 n-6**	0.013	0.023
				**18:4 n-3**	0.003	0.004
				**20:5 n-3**	-0.011	0.033
				**22:6 n-3**	0.072	0.090

(continued Table S8.)

**Fig. 2D**	**Eigenvalue**	**% variance**	**% Cumulative variance**	**Eigenvectors**	**PC 1**	**PC 2**
**PC 1**	55.542	80.623	80.623	**14:0**	-0.111	0.210
**PC 2**	9.450	13.717	94.340	**16:0**	-0.344	0.405
**PC 3**	2.720	3.948	98.288	**18:0**	-0.065	-0.781
**PC 4**	0.754	1.095	99.383	**20:0**	0.000	0.000
**PC 5**	0.267	0.387	99.770	**22:0**	-0.015	0.094
**PC 6**	0.095	0.137	99.907	**16:1 n-7**	0.035	-0.006
**PC 7**	0.039	0.057	99.964	**20:2 n-9**	0.009	0.011
**PC 8**	0.022	0.032	99.996	**22:1 n-9**	-0.005	0.054
**PC 9**	0.003	0.004	100.000	**18:1 n-9**	-0.058	0.061
				**18:2 n-6**	0.052	0.066
				**18:3 n-6**	0.316	0.120
				**20:3 n-6**	0.000	0.000
				**20:4 n-6**	0.000	0.000
				**22:4 n-6**	0.000	0.000
				**18:4 n-3**	0.032	0.000
				**20:5 n-3**	0.074	-0.073
				**22:6 n-3**	0.081	-0.160

Table S9. The PCA eigenvalue scores of temporal contribution of LBs individual lipids.

**Fig. 4A**	**Eigenvalue**	**% variance**	**% Cumulative variance**	**Eigenvectors**	**PC 1**	**PC 2**
**PC 1**	466.710	82.342	82.342	**14:0**	0.040	-0.059
**PC 2**	57.080	10.071	92.413	**16:0**	-0.281	0.016
**PC 3**	25.813	4.554	96.967	**18:0**	-0.114	-0.166
**PC 4**	6.917	1.220	98.188	**20:0**	-0.046	-0.009
**PC 5**	4.701	0.829	99.017	**22:0**	0.013	-0.064
**PC 6**	2.095	0.370	99.387	**16:1 n-7**	0.043	0.036
**PC 7**	1.488	0.263	99.649	**20:2 n-9**	0.009	-0.017
**PC 8**	0.618	0.109	99.758	**22:1 n-9**	-0.024	0.105
**PC 9**	0.589	0.104	99.862	**18:1 n-9**	0.018	0.512
**PC 10**	0.494	0.087	99.949	**18:2 n-6**	-0.075	-0.054
**PC 11**	0.116	0.020	99.970	**18:3 n-6**	0.408	-0.243
**PC 12**	0.090	0.016	99.986	**20:3 n-6**	-0.059	0.068
**PC 13**	0.066	0.012	99.997	**20:4 n-6**	-0.309	0.076
**PC 14**	0.015	0.003	100.000	**22:4 n-6**	-0.088	-0.004
				**18:4 n-3**	0.082	-0.052
				**20:5 n-3**	0.257	-0.178
				**22:6 n-3**	0.127	0.032

**Fig. 4B**	**Eigenvalue**	**% variance**	**% Cumulative variance**	**Eigenvectors**	**PC 1**	**PC 2**
**PC 1**	427.421	69.219	69.219	**14:0**	-0.009	0.053
**PC 2**	129.718	21.007	90.226	**16:0**	-0.194	-0.021
**PC 3**	32.834	5.317	95.543	**18:0**	-0.351	-0.166
**PC 4**	12.739	2.063	97.606	**20:0**	0.000	0.000
**PC 5**	7.156	1.159	98.765	**22:0**	0.000	0.000
**PC 6**	4.094	0.663	99.428	**16:1 n-7**	0.011	-0.035
**PC 7**	1.922	0.311	99.740	**20:2 n-9**	0.026	0.130
**PC 8**	0.957	0.155	99.895	**22:1 n-9**	0.060	0.308
**PC 9**	0.454	0.074	99.968	**18:1 n-9**	0.000	0.000
**PC 10**	0.177	0.029	99.997	**18:2 n-6**	0.088	0.106
**PC 11**	0.014	0.002	100.000	**18:3 n-6**	0.245	-0.641
				**20:3 n-6**	0.000	0.009
				**20:4 n-6**	0.035	0.132
				**22:4 n-6**	0.000	0.000
				**18:4 n-3**	0.000	0.000
				**20:5 n-3**	0.023	0.002
				**22:6 n-3**	0.065	0.125

**Fig. 4C**	**Eigenvalue**	**% variance**	**% Cumulative variance**	**Eigenvectors**	**PC 1**	**PC 2**
**PC 1**	962.225	82.173	82.173	**14:0**	-0.002	-0.086
**PC 2**	154.014	13.153	95.326	**16:0**	-0.180	-0.093
**PC 3**	26.195	2.237	97.563	**18:0**	-0.352	0.016
**PC 4**	13.731	1.173	98.736	**20:0**	-0.009	-0.002
**PC 5**	5.853	0.500	99.235	**22:0**	-0.016	-0.030
**PC 6**	3.841	0.328	99.563	**16:1 n-7**	0.033	0.091
**PC 7**	1.961	0.167	99.731	**20:2 n-9**	-0.008	0.274
**PC 8**	1.535	0.131	99.862	**22:1 n-9**	0.000	0.000
**PC 9**	0.884	0.075	99.937	**18:1 n-9**	0.009	0.203
**PC 10**	0.494	0.042	99.980	**18:2 n-6**	0.042	0.067
**PC 11**	0.211	0.018	99.998	**18:3 n-6**	0.226	-0.506
**PC 12**	0.020	0.002	100.000	**20:3 n-6**	0.000	0.000
				**20:4 n-6**	0.012	0.334
				**22:4 n-6**	-0.012	0.073
				**18:4 n-3**	0.047	-0.101
				**20:5 n-3**	0.113	-0.197
				**22:6 n-3**	0.097	-0.043

(continued Table S9.)

Fig. 4D	Eigenvalue	% variance	% Cumulative variance	Eigenvectors	PC 1	PC 2
**PC 1**	530.430	68.389	68.389	**14:0**	0.005	-0.087
**PC 2**	165.689	21.363	89.752	**16:0**	-0.069	-0.304
**PC 3**	46.566	6.004	95.756	**18:0**	-0.214	-0.099
**PC 4**	17.713	2.284	98.040	**20:0**	-0.002	0.013
**PC 5**	5.367	0.692	98.732	**22:0**	0.028	-0.009
**PC 6**	3.583	0.462	99.194	**16:1 n-7**	0.007	-0.004
**PC 7**	3.421	0.441	99.635	**20:2 n-9**	0.021	0.007
**PC 8**	1.973	0.254	99.889	**22:1 n-9**	-0.209	0.002
**PC 9**	0.533	0.069	99.958	**18:1 n-9**	0.021	0.014
**PC 10**	0.117	0.015	99.973	**18:2 n-6**	0.022	0.002
**PC 11**	0.105	0.014	99.987	**18:3 n-6**	0.314	-0.109
**PC 12**	0.062	0.008	99.994	**20:3 n-6**	-0.003	0.049
**PC 13**	0.024	0.003	99.998	**20:4 n-6**	-0.398	0.384
**PC 14**	0.008	0.001	100.000	**22:4 n-6**	-0.095	0.150
				**18:4 n-3**	0.094	-0.044
				**20:5 n-3**	0.302	-0.018
				**22:6 n-3**	0.176	0.054

Fig. 4E	Eigenvalue	% variance	% Cumulative variance	Eigenvectors	PC 1	PC 2
**PC 1**	257.842	82.771	82.771	**R=C14/R'=C16**	0.097	0.157
**PC 2**	47.804	15.346	98.117	**R=C16/R'=C16**	0.698	-0.417
**PC 3**	5.866	1.883	100.000	**R=C18/R'=C16**	-0.091	0.750
				**R=C18:1/R'=C16**	-0.704	-0.489

Table S10. The PCA eigenvalue scores of inter-compartmental lipid transportation.

**Fig. 5A**	**Eigenvalue**	**% variance**	**% Cumulative variance**	**Eigenvectors**	**PC 1**	**PC 2**
**PC 1**	700.199	79.612	79.612	**14:0**	-0.010	0.017
**PC 2**	63.448	7.214	86.826	**16:0**	-0.237	-0.083
**PC 3**	51.741	5.883	92.709	**18:0**	-0.309	-0.029
**PC 4**	34.118	3.879	96.588	**20:0**	-0.001	-0.032
**PC 5**	10.966	1.247	97.835	**22:0**	-0.002	-0.034
**PC 6**	5.633	0.640	98.475	**16:1 n-7**	0.010	0.072
**PC 7**	4.713	0.536	99.011	**20:2 n-9**	0.006	-0.019
**PC 8**	3.245	0.369	99.380	**22:1 n-9**	0.051	0.124
**PC 9**	1.762	0.200	99.581	**18:1 n-9**	-0.002	0.469
**PC 10**	1.586	0.180	99.761	**18:2 n-6**	0.004	0.223
**PC 11**	0.729	0.083	99.844	**18:3 n-6**	0.014	-0.025
**PC 12**	0.648	0.074	99.918	**20:3 n-6**	0.035	-0.082
**PC 13**	0.526	0.060	99.977	**20:4 n-6**	0.236	-0.339
**PC 14**	0.117	0.013	99.991	**22:4 n-6**	0.070	-0.160
**PC 15**	0.0497	0.0056	99.996	**18:4 n-3**	0.002	-0.017
**PC 16**	0.03195	0.00363	100.000	**20:5 n-3**	0.047	-0.082
				**22:6 n-3**	0.086	-0.002

**Fig. 5B**	**Eigenvalue**	**% variance**	**% Cumulative variance**	**Eigenvectors**	**PC 1**	**PC 2**
**PC 1**	851.843	76.784	76.784	**14:0**	-0.033	-0.088
**PC 2**	111.391	10.041	86.825	**16:0**	-0.166	-0.120
**PC 3**	59.893	5.399	92.224	**18:0**	-0.345	0.067
**PC 4**	46.895	4.227	96.451	**20:0**	-0.002	0.032
**PC 5**	16.584	1.495	97.946	**22:0**	0.005	0.007
**PC 6**	7.207	0.650	98.595	**16:1 n-7**	0.004	-0.029
**PC 7**	5.861	0.528	99.124	**20:2 n-9**	0.011	-0.001
**PC 8**	3.881	0.350	99.473	**22:1 n-9**	-0.031	0.051
**PC 9**	2.525	0.228	99.701	**18:1 n-9**	-0.046	0.067
**PC 10**	1.541	0.139	99.840	**18:2 n-6**	0.007	-0.005
**PC 11**	0.740	0.067	99.907	**18:3 n-6**	0.168	-0.517
**PC 12**	0.524	0.047	99.954	**20:3 n-6**	0.021	0.161
**PC 13**	0.308	0.028	99.982	**20:4 n-6**	0.065	0.524
**PC 14**	0.099	0.009	99.991	**22:4 n-6**	0.023	0.195
**PC 15**	0.0612	0.0055	99.996	**18:4 n-3**	0.043	-0.099
**PC 16**	0.04176	0.00376	100.000	**20:5 n-3**	0.150	-0.255
				**22:6 n-3**	0.127	0.007

**Fig. 5C**	**Eigenvalue**	**% variance**	**% Cumulative variance**	**Eigenvectors**	**PC 1**	**PC 2**
**PC 1**	732.826	68.008	68.008	**14:0**	-0.024	-0.060
**PC 2**	193.036	17.914	85.922	**16:0**	-0.212	-0.057
**PC 3**	56.685	5.261	91.183	**18:0**	-0.327	0.006
**PC 4**	46.573	4.322	95.505	**20:0**	-0.003	0.001
**PC 5**	23.554	2.186	97.691	**22:0**	0.014	-0.026
**PC 6**	9.694	0.900	98.590	**16:1 n-7**	0.010	0.013
**PC 7**	5.752	0.534	99.124	**20:2 n-9**	0.008	-0.013
**PC 8**	3.542	0.329	99.453	**22:1 n-9**	0.017	0.300
**PC 9**	2.259	0.210	99.662	**18:1 n-9**	-0.045	-0.018
**PC 10**	1.618	0.150	99.812	**18:2 n-6**	0.012	-0.009
**PC 11**	1.266	0.118	99.930	**18:3 n-6**	0.197	-0.374
**PC 12**	0.448	0.042	99.971	**20:3 n-6**	-0.008	0.016
**PC 13**	0.209	0.019	99.991	**20:4 n-6**	0.043	0.533
**PC 14**	0.042	0.004	99.995	**22:4 n-6**	0.011	0.124
**PC 15**	0.0328	0.0030	99.998	**18:4 n-3**	0.048	-0.080
**PC 16**	0.02118	0.00197	100.000	**20:5 n-3**	0.150	-0.249
				**22:6 n-3**	0.109	-0.106

Table S11. The PCA eigenvalue scores of individual lipid species in three cellular compartments.

**Fig. S1**	**Eigenvalue**	**% variance**	**% Cumulative variance**	**Eigenvectors**	**PC 1**	**PC 2**
**PC 1**	821.755	86.239	86.239	**14:0**	-0.019	0.030
**PC 2**	73.398	7.703	93.942	**16:0**	-0.200	0.108
**PC 3**	24.334	2.554	96.496	**18:0**	-0.327	-0.210
**PC 4**	15.019	1.576	98.072	**20:0**	0.002	-0.043
**PC 5**	6.601	0.693	98.764	**22:0**	-0.004	-0.060
**PC 6**	3.550	0.373	99.137	**16:1 n-7**	0.000	0.000
**PC 7**	3.449	0.362	99.499	**20:2 n-9**	0.011	-0.026
**PC 8**	2.106	0.221	99.720	**22:1 n-9**	-0.031	0.184
**PC 9**	1.402	0.147	99.867	**18:1 n-9**	-0.003	0.447
**PC 10**	0.616	0.065	99.932	**18:2 n-6**	0.000	0.226
**PC 11**	0.291	0.031	99.962	**18:3 n-6**	0.023	-0.038
**PC 12**	0.220	0.023	99.985	**20:3 n-6**	0.064	-0.047
**PC 13**	0.064	0.007	99.992	**20:4 n-6**	0.208	-0.262
**PC 14**	0.038	0.004	99.996	**22:4 n-6**	0.074	-0.182
**PC 15**	0.0334	0.0035	100.000	**18:4 n-3**	0.006	-0.001
				**20:5 n-3**	0.076	-0.115
				**22:6 n-3**	0.120	-0.009

**Fig. S2**	**Eigenvalue**	**% variance**	**% Cumulative variance**	**Eigenvectors**	**PC 1**	**PC 2**
**PC 1**	637.681	80.706	80.706	**14:0**	0.001	-0.024
**PC 2**	75.079	9.502	90.208	**16:0**	-0.278	-0.157
**PC 3**	41.889	5.302	95.510	**18:0**	-0.269	-0.008
**PC 4**	17.114	2.166	97.676	**20:0**	-0.005	0.000
**PC 5**	6.634	0.840	98.515	**22:0**	0.000	0.000
**PC 6**	4.795	0.607	99.122	**16:1 n-7**	0.023	0.110
**PC 7**	3.024	0.383	99.505	**20:2 n-9**	0.000	0.000
**PC 8**	2.079	0.263	99.768	**22:1 n-9**	0.160	-0.255
**PC 9**	0.800	0.101	99.869	**18:1 n-9**	-0.006	0.481
**PC 10**	0.608	0.077	99.946	**18:2 n-6**	0.006	0.217
**PC 11**	0.256	0.032	99.978	**18:3 n-6**	0.000	0.060
**PC 12**	0.112	0.014	99.993	**20:3 n-6**	-0.008	0.073
**PC 13**	0.054	0.007	99.999	**20:4 n-6**	0.272	-0.542
**PC 14**	0.006	0.001	100.000	**22:4 n-6**	0.064	-0.132
				**18:4 n-3**	-0.004	0.010
				**20:5 n-3**	0.007	0.009
				**22:6 n-3**	0.036	0.158

**Fig. S3**	**Eigenvalue**	**% variance**	**% Cumulative variance**	**Eigenvectors**	**PC 1**	**PC 2**
**PC 1**	908.959	84.887	84.887	**14:0**	-0.063	-0.122
**PC 2**	105.255	9.830	94.717	**16:0**	-0.139	-0.025
**PC 3**	30.477	2.846	97.563	**18:0**	-0.337	0.138
**PC 4**	15.108	1.411	98.974	**20:0**	0.000	0.000
**PC 5**	6.419	0.599	99.573	**22:0**	0.018	-0.035
**PC 6**	2.090	0.195	99.768	**16:1 n-7**	0.000	0.027
**PC 7**	1.055	0.099	99.867	**20:2 n-9**	0.011	-0.026
**PC 8**	0.721	0.067	99.934	**22:1 n-9**	-0.018	-0.027
**PC 9**	0.422	0.039	99.974	**18:1 n-9**	-0.084	-0.045
**PC 10**	0.173	0.016	99.990	**18:2 n-6**	0.013	0.101
**PC 11**	0.071	0.007	99.997	**18:3 n-6**	0.210	0.499
**PC 12**	0.040	0.004	100.000	**20:3 n-6**	0.000	0.000
				**20:4 n-6**	0.000	0.000
				**22:4 n-6**	0.000	0.000
				**18:4 n-3**	0.066	-0.080
				**20:5 n-3**	0.188	-0.245
				**22:6 n-3**	0.136	-0.159
